# Genetic prediction of the causal relationship between schizophrenia and tumors: a Mendelian randomized study

**DOI:** 10.3389/fonc.2024.1321445

**Published:** 2024-02-16

**Authors:** Xintong Zhou, Qi Liu, Shihan Liu, Liquan Wang, Zhongli Sun, Changgang Sun, Xiangning Cui

**Affiliations:** ^1^ College of First Clinical Medicine, Shandong University of Traditional Chinese Medicine, Jinan, China; ^2^ Department of Otorhinolaryngology, the Second Affiliated Hospital of Chongqing Medical University, Chongqing, China; ^3^ Department of Thyroid and Breast Surgery, Weifang People’s Hospital, Weifang, China; ^4^ College of Traditional Chinese Medicine, Weifang Medical University, Weifang, China; ^5^ Department of Oncology, Weifang Traditional Chinese Hospital, Weifang, China; ^6^ Department of Cardiovascular, Guang’anmen Hospital, China Academy of Chinese Medical Sciences, Beijing, China

**Keywords:** schizophrenia, cancer, mendelian randomization, causality, GWAS

## Abstract

**Background:**

Patients with schizophrenia are at a higher risk of developing cancer. However, the causal relationship between schizophrenia and different tumor types remains unclear.

**Methods:**

Using a two-sample, two-way Mendelian randomization method, we used publicly available genome-wide association analysis (GWAS) aggregate data to study the causal relationship between schizophrenia and different cancer risk factors. These tumors included lung adenocarcinoma, lung squamous cell carcinoma, small-cell lung cancer, gastric cancer, alcohol-related hepatocellular cancer, tumors involving the lungs, breast, thyroid gland, pancreas, prostate, ovaries and cervix, endometrium, colon and colorectum, and bladder. We used the inverse variance weighting (IVW) method to determine the causal relationship between schizophrenia and different tumor risk factors. In addition, we conducted a sensitivity test to evaluate the effectiveness of the causality.

**Results:**

After adjusting for heterogeneity, evidence of a causal relationship between schizophrenia and lung cancer risk was observed (odds ratio [OR]=1.001, 95% confidence interval [CI], 1.000–1.001; *P*=0.0155). In the sensitivity analysis, the causal effect of schizophrenia on the risk of lung cancer was consistent in both direction and degree. However, no evidence of causality or reverse causality between schizophrenia and other tumors was found.

**Conclusion:**

This study elucidated a causal relationship between the genetic predictors of schizophrenia and the risk of lung cancer, thereby providing a basis for the prevention, pathogenesis, and treatment of schizophrenia in patients with lung cancer.

## 1 Introduction

Schizophrenia is a serious mental illness that affects the thinking, behavior, and emotions of patients (1). Recent studies have demonstrated a complex relationship between schizophrenia and tumor incidence (2).

Symptoms of schizophrenia include hallucinations, delusions, and confusion (3, 4), whereas tumors are pathological changes caused by excessive cell growth. Although they seem unrelated, the relationship between them may be more complicated than anticipated. However, whether there is a causal relationship between schizophrenia and tumors remains controversial. Some observational studies have suggested a causal relationship between schizophrenia and tumor risk (5), whereas others have not (6). In addition, the incidence of tumors in patients with schizophrenia differs from that in the general population. Patients with schizophrenia may have a lower cancer incidence (7); however, this does not imply people with schizophrenia will not develop tumors but that the risk is relatively low. In contrast, a significant difference was observed in the overall incidence of cancer among people diagnosed with mental disorders, and tumors are often diagnosed at an advanced stage in patients with mental disorders (8). Patients with schizophrenia may be at increased risk of developing certain types of tumors following antipsychotic treatment (9), which could be attributed to lifestyle, drug therapy, and genetic factors.

Although the relationship between schizophrenia and tumors is not fully understood, this possible link is of considerable importance for the research and treatment of schizophrenia. Understanding this relationship may help us better understand the pathological mechanisms of schizophrenia and provide new implications for future treatment strategies. The relationship between schizophrenia and tumors is complex and delicate (2). Further research is needed to explore and understand this relationship to provide a scientific basis for the prevention and treatment of schizophrenia and tumors.

When observational studies explore the causal association between schizophrenia and cancer, the level of evidence for causal inference is often low due to confounding factors such as smoking, body mass index, socioeconomic status (SES), antipsychotic drugs, and genetics (10–12). **Table 1** summarized the common confounding factors affecting schizophrenia and tumors. Among the diverse epidemiological research methods, randomized controlled trials are the strongest research design in clinical trials because of their randomness, control, and blindness. Randomized controlled trials are the “gold standard” to verify causality (27). However, because the research and implementation cost too much time, money and manpower; Strict inclusion criteria may lead to a decrease in the representativeness of the test population to the target population. The standard interventions used are not completely consistent with clinical practice; Limited sample size and short follow-up lead to inadequate detection of rare adverse events. These limitations pose challenges in extrapolating the findings of Randomized Controlled Trial (RCT) to practical clinical applications. In addition, for rare and life-threatening diseases that lack effective treatments, routine RCTS may be difficult to implement, require high time costs, or may raise ethical concerns. Therefore, randomized controlled trials face various challenges in clinical practice (28–30).

**Table 1:** Common confounding factors affecting schizophrenia and tumors.

Confounding factor	Disease	Effect	Reference
Smoke	Schizophrenia	A link between the physiological effects of nicotine and the neurobiological disturbances in schizophrenia	(13)
	Cancer	Carcinogens in cigarette smoke cause molecular changes in lung cancer patients	(14)
Parasomnia	Schizophrenia	Schizophrenia has long been associated with abnormalities in circadian rhythms and sleep	(15)
	Cancer	Insomnia has an impact on lung adenocarcinoma risk	(16)
Socioeconomic status	Schizophrenia	Increased schizophrenia prevalence in countries with low levels of income inequality	(17)
	Cancer	It is often reported in the literature that breast cancer is more deadly in low to middle income countries due to younger age at diagnosis and more aggressive biology compared with high income countries	(18)
Antipsychotics	Schizophrenia	Clozapine is an effective treatment for schizophrenia in children and adolescents	(19)
	Cancer	Long-term exposure to prolactin-increasing, but not to prolactin-sparing, antipsychotics is significantly associated with increased odds of breast cancer	(20)
Body Mass Index	Schizophrenia	Schizophrenia is frequently associated with obesity	(21)
	Cancer	Increased BMI is associated with increased risk of common and less common malignancies	(22)
Heredity	Schizophrenia	A clear genetic susceptibility is present in schizophrenia	(23)
	Cancer	Family history is an important risk factor for breast cancer incidence	(24)
Air pollution	Schizophrenia	Air pollutants increase the risk of schizophrenia	(25)
	Cancer	Positive associations between air pollution exposure and bladder cancer mortality and kidney cancer incidence	(26)

Mendelian randomization (MR) is a statistical method for evaluating etiological inferences in epidemiological studies. MR is based on genome-wide sequencing data and uses genetic variations with strong correlations to exposure factors as tool variables to assess causal relationships between exposure factors and clinical outcomes (31). During meiosis, alleles are randomly separated; therefore, MR can reduce deviations caused by confounding factors (32).

In this study, a large-scale genome-wide association analysis (GWAS) dataset was used to conduct a two-sample MR study to explore the causal relationship between schizophrenia and the risk of multiple cancers. These tumors included lung adenocarcinoma, lung squamous cell carcinoma, small-cell lung cancer, gastric cancer, alcohol-related hepatocellular cancer, tumors involving the lungs, breast, thyroid gland, pancreas, prostate, ovaries and cervix, endometrium, colon and colorectum, and bladder. This study will help reveal the causal relationship between schizophrenia and cancer, which may aid in prevention and treatment.

## 2 Materials and methods

### 2.1 Data sources

We used publicly available schizophrenia (52017 cases and 75889 controls, European origin) GWAS data (33) and selected a statistically significant Single Nucleotide Polymorphism (SNP) (p < 5 × 10-8) that was closely related to schizophrenia from the GWAS. We used publicly available oncology data from the MRC Integrative Epidemiology Unit (IEU, https://gwas.mrcieu.ac.uk//; accessed on September 17, 2023).

Because the data in this study were obtained from a public database, ethical approval from the participants was not required.

### 2.2 *P* value and F-statistics calculation

We used F-statistics to evaluate the statistical strength. The F-statistics are equal to ((N-k-1)/k) × (R2/(1-R2)), where N and k represent the sample size and number of SNP, respectively (34). An F-statistic < 10 indicates that the genetic variation used is a weak tool variable that may contribute to a bias. We determined the variance in phenotype explained by each instrument by R2: R2 = [2 × EAF × (1-EAF) × (β) 2]/[(2 × EAF × (1-EAF) × (β) 2) + (2 × EAF × (1-EAF) × N × se (β) 2)], where EAF is the effect allele frequency, β is the effect quantity, N is the sample size, and Se (β) is the standard error of the effect quantity. We used R2 and F-statistics to evaluate the statistical power of each tool variable. **Supplementary Table S1** shows the number of MR statistical powers and valid tool variables for each pair.

### 2.3 Statistical analysis

**Figure 1:**
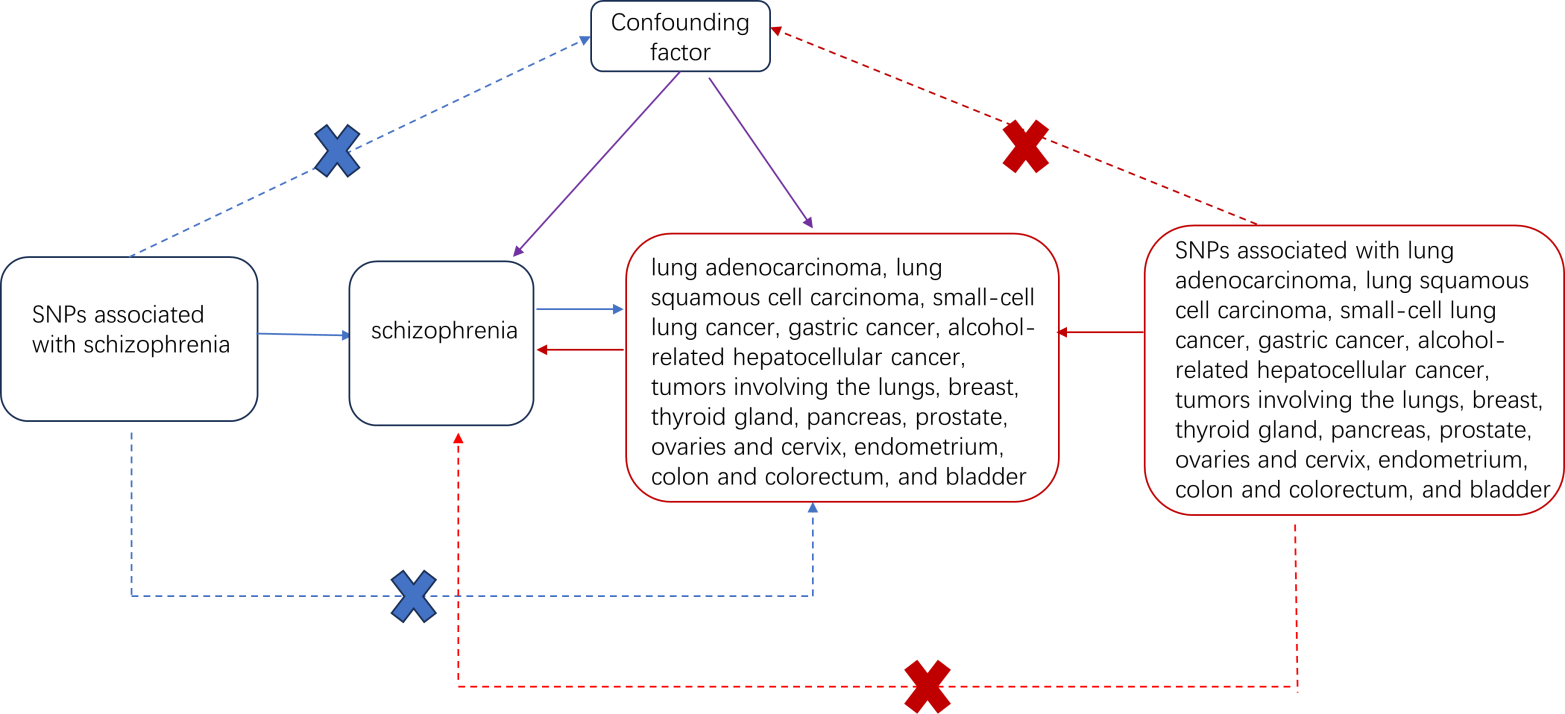
Two-sample Mendelian randomized study design for schizophrenia and different cancers. Solid blue lines indicate the association between tool variables (SNP) and exposure (schizophrenia) and between exposure and outcome (different types of tumors). The red solid line indicates the correlation of reverse causality. The dotted line with crossover indicates that this association conforms to two basic assumptions of Mendelian randomization: 1. Genetic variation (SNP) is independent of the confounding factor between exposure and outcome 2. Genetic variation affects the results only through exposure.

The causal relationship between exposure (schizophrenia) and outcome (tumor) was determined using two-sample Mendelian randomization (35). **Figure 1** illustrates the study process. The inverse variance weighting (IVW) method was used to estimate the causal relationship between exposure and outcome (36). This method ignores the existence of intercept terms and fits the reciprocal of the outcome variance as a weight; therefore, it can reliably estimate the impact of exposure on the outcome under the condition of heterogeneity between tool variables (37). The results are presented as odds ratios (OR) and 95% confidence intervals (CI). IVW is the most effective MR method; however, it assumes that all tool variables are valid. If the horizontal multiplicity is not zero, a bias exists. The weighted median method used most SNPs to determine causality (38). Therefore, we conducted a sensitivity analysis to ensure the accuracy of the causal effect results, including the leave-one-out sensitivity test, heterogeneity test, and pleiotropy test. The main purpose of the leave-one-out sensitivity test was to calculate the MR results of the remaining instrumental variable (IV) after eliminating it. If there is a substantial difference between the estimated MR and the summary results of other IVs after excluding a certain IV, the MR results are sensitive to the IV. The main purpose of the heterogeneity test was to examine the differences between the different IVs. Moreover, if there are considerable differences between different IVs, the heterogeneity of these IVs is large. The multiplicity test mainly tests whether there is horizontal multiplicity in multiple IV. The MR–Egger regression intercept is commonly used to express horizontal multiplicity if the intercept is not zero. In addition, the MR-PRESSO package is a commonly used R packet for testing horizontal multiplicity. MR analysis was performed using the R package “Two Sample MR” (version 0.5.7) in R (version 4.3.1).

## 3 Results

### 3.1 Causality between schizophrenia and tumor

**Figure 2:**
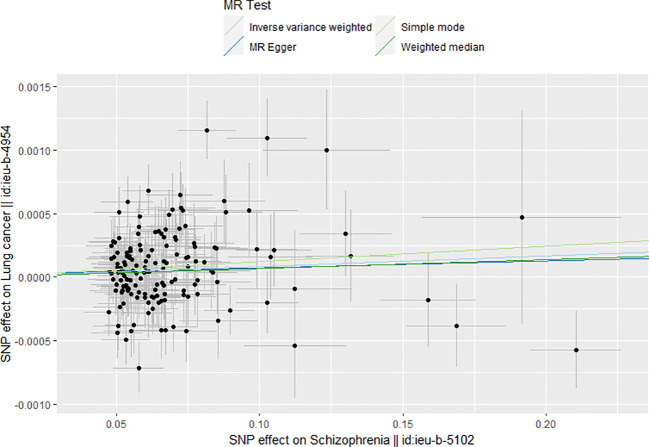
MR Scatter plot.

**Figure 3:**
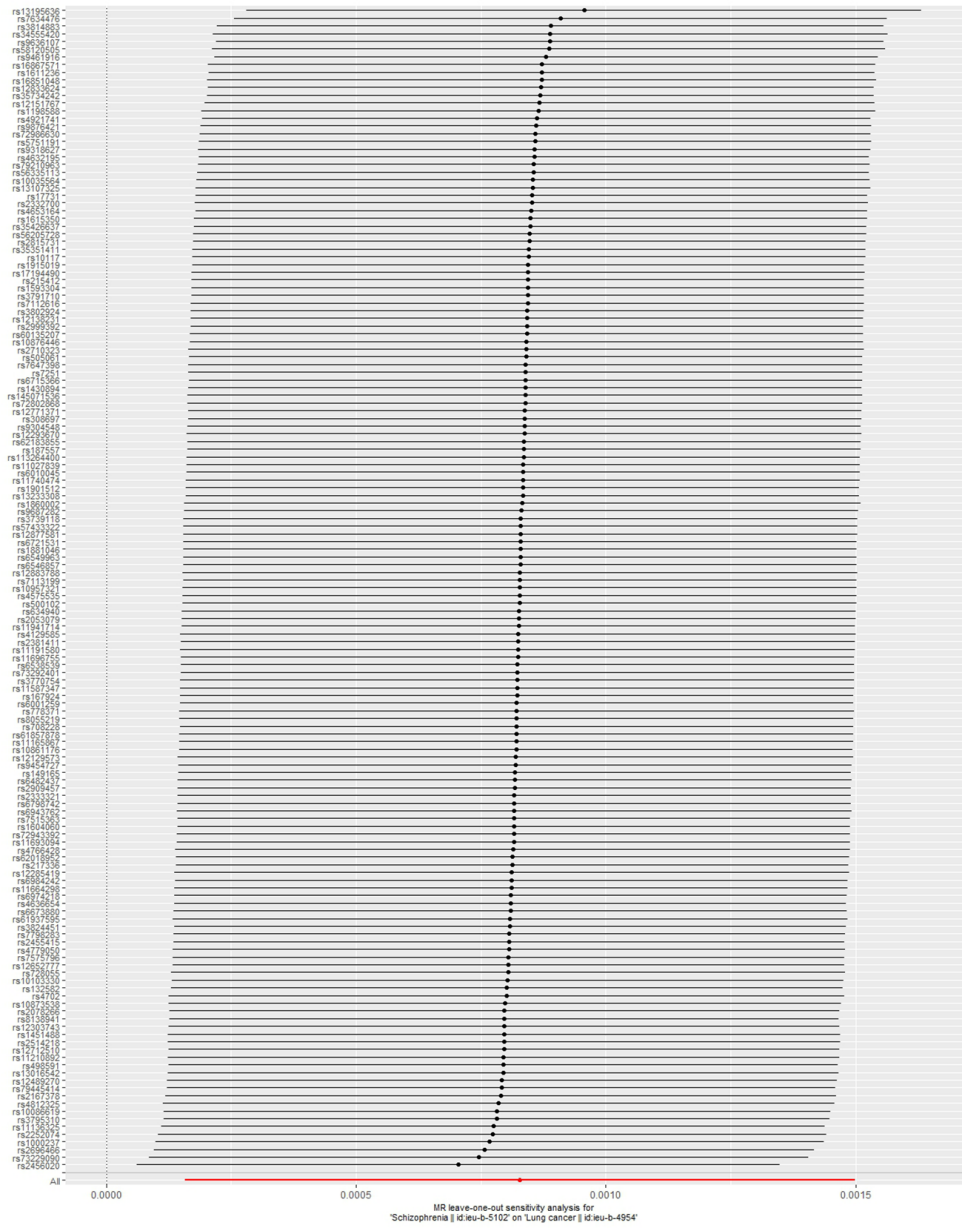
MR leave-one-out sensitivity analysis for Schizophrenia on Lung cancer.

**Table 2** shows the results of the MR analysis of different tumors in patients with schizophrenia, which shows that schizophrenia is associated with an increased risk of lung cancer (**Figure 2**). Lung cancer (OR=1.001, 95% CI: 1.000–1.001, *P*=0.0155), lung adenocarcinoma (OR=1.041, 95% CI: 0.976–1.110, *P*=0.2250), lung squamous cell carcinoma (OR=1.081, 95% CI: 0.990–1.179, *P*=0.0813), small cell lung cancer (OR=1.000, 95% CI: 0.839–1.193, *P*=0.9968), breast cancer (OR=1.000, 95% CI: 0.999–1.001, *P*=0.3448), thyroid cancer (OR=0.950, 95% CI:0.849–1.061, P=0.3617), gastric cancer (OR=0.998, 95% CI: 0.947–1.052, *P*=0.9533), pancreatic cancer (OR=1.037, 95% CI: 0.931–1.155, *P*=0.5096), alcohol-related hepatocellular carcinoma (OR=1.010, 95% CI: 0.806–1.265, *P*=0.9331), prostate cancer (OR=1.000, 95% CI: 0.999–1.001, *P*=0.2712), ovarian cancer (OR=1.049, 95% CI: 0.964–1.142). Cervical cancer (OR=1.000, 95% CI: 0.999–1.000, *P*=0.4674), endometrial cancer (OR=1.000, 95% CI: 0.999–1.000, *P*=0.2516), colon cancer (OR=1.000, 95% CI: 0.999–1.000, *P*=0.9582), colorectal cancer (OR=1.000, 95% CI: 0.961–1.040, *P*=0.9939), bladder cancer (OR=1.000, 95% CI: 0.999–1.000, *P*=0.7360). **Table 2** shows that the heterogeneity tests of lung cancer, lung adenocarcinoma, lung squamous carcinoma and breast cancer are all much less than 0.05. This suggests that the results may be heterogeneous. We used the random-effect method to estimate the MR effect. From the results of the random-effect model, it is not difficult to see that there is a causal relationship between schizophrenia and lung cancer (*p*val < 0.05), and schizophrenia increases the risk of lung cancer (b > 0). This may suggest that there is a statistical causal relationship between schizophrenia and lung cancer (*P*= 0.0155). In the pleiotropy test, the *P* values of all outcomes are greater than 0.05. There is no statistical difference, so it can be considered that there is no horizontal pleiotropy. So the genetic variation in this study does not affect the outcome variables other than exposure. The leave-one-out sensitivity analysis in **Figure 3** shows that all beta values are in the same direction, which means that the positive causal relationship between exposure and outcome still exists after any single SNP is removed.

**Table 2:** Results of MR analysis of different tumors in schizophrenia.

Datasets Num	Cancer Type	MR Results (IVW- *P* value)	OR (95% CI)	Heterogeneity Statistics	Pleiotropy Test
ieu-b-4954	Lung cancer	0.0155	1.001 (1.000–1.001)	4.225711e-06	0.82
ebi-a-GCST004744	Lung adenocarcinoma	0.2250	1.041 (0.976–1.110)	4.881943e-12	0.94
ebi-a-GCST004750	Lung squamous carcinoma	0.0813	1.081 (0.990–1.179)	6.341086e-25	0.68
finn-b-C3_NSCLC_ADENO_EXALLC	Small cell lung cancer	0.9968	1.000 (0.839–1.193)	0.16	0.68
ukb-b-16890	Breast cancer	0.3448	1.000 (0.999–1.001)	0.008	0.23
ebi-a-GCST90018929	Thyroid cancer	0.3617	0.950 (0.849–1.061)	0.37	0.21
ebi-a-GCST90018849	Gastric cancer	0.9533	0.998 (0.947–1.052)	0.49	0.17
ebi-a-GCST90018893	Pancreatic cancer	0.5096	1.037 (0.931–1.155)	0.10	0.10
ebi-a-GCST90092003	Alcohol-related hepatocellular carcinoma	0.9331	1.010 (0.806–1.265)	0.09	0.07
ukb-b-13348	Prostate cancer	0.2712	1.000 (0.999–1.001)	0.18	0.94
ebi-a-GCST90018888	Ovarian cancer	0.2685	1.049 (0.964–1.142)	0.90	0.68
ukb-b-8777	Cervical cancer	0.4674	1.000 (0.999–1.000)	0.02	0.78
ukb-b-13545	Endometrial cancer	0.2516	1.000 (0.999–1.000)	0.74	0.33
ukb-b-20145	Colon cancer	0.9582	1.000 (0.999–1.000)	0.61	0.24
ebi-a-GCST90018808	Colorectal cancer	0.9939	1.000 (0.961–1.040)	0.13	0.09
ukb-b-8193	Bladder cancer	0.7360	1.000 (0.999–1.000)	0.24	0.05

### 3.2 Causal relationship between tumor and schizophrenia

To analyze the causal effects of different tumors on the risk of schizophrenia, we first identified SNPs that are closely related to different tumors. Among the 16 tumor types, only 6 had a strong correlation SNP, 6 had no strong correlation SNP, and 4 had <5 SNP. Therefore, we could not conduct an MR analysis of these 10 tumors. The IVW-*P* value of prostate cancer was 0.008 in the reverse MR analysis; however, the beta value of the MR–Egger was not consistent with the direction of IVW in further MR analysis, and the result was invalid. Therefore, we found no effect of tumors on the risk of schizophrenia, indicating that almost all tumors had no significant effect on the risk of schizophrenia (*P* > 0.05; **Table 3**).

**Table 3:** Results of MR analysis of tumor in schizophrenia.

Cancer Type	SNP	MR Results (IVW-*P* value)	OR (95% CI)
Lung cancer	2		
Lung adenocarcinoma	14	0.36	1.034 (0.963–1.110)
Lung squamous carcinoma	7	0.26	0.889 (0.723–1.091)
Small cell lung cancer	0		
Breast cancer	19	0.63	0.528 (3.966–7.026)
Thyroid cancer	0		
Gastric cancer	10	0.28	1.086 (0.936–1.261)
Pancreatic cancer	0		
Alcohol-related hepatocellular carcinoma	2		
Prostate cancer	18	0.008	0.01 (3.051e-04–3.052e-01)
Ovarian cancer	0		
Cervical cancer	1		
Endometrial cancer	0		
Colon cancer	0		
Colorectal cancer	30	0.84	1.004 (0.964–1.046)
Bladder cancer	2		

## 4 Discussion

To date, the possibility of a causal relationship between schizophrenia and tumor incidence remains controversial. Cohort studies by some scholars have shown that there is no significant difference in the incidence rates of colorectal cancer, breast cancer, lung cancer, and all common cancers between patients with schizophrenia and patients without schizophrenia (39, 40). However, some scholars have also found in cohort studies that, under the influence of different confounding factors, the incidence rates of schizophrenia and different cancers in different groups have completely different effects. For example, under the influence of the confounding factor of smoking, the incidence of lung cancer in male patients with schizophrenia is reduced (41), while the incidence of lung cancer and breast cancer in women is increased (42). This shows that confounding factors do have a significant impact between the two, and the way of impact is uncertain. The relationship between schizophrenia and cancer is controversial, possibly owing to research design and confounding factors such as smoking or diet, antipsychotics, and different cancer screening and treatment methods (11, 12). Moreover, cancer is a geriatric disease, and the life expectancy of patients with schizophrenia is shortened by 10–25 years (43). The advantage of MR is to control confounding factors, so that we can get more reliable and accurate results between exposure and outcome at the genetic level. This study examined the causal relationship between genetically predicted schizophrenia and the risk of various cancers, including lung adenocarcinoma, lung squamous cell carcinoma, small-cell lung cancer, gastric cancer, alcohol-related hepatocellular cancer, tumors involving the lungs, breast, thyroid gland, pancreas, prostate, ovaries and cervix, endometrium, colon and colorectum, and bladder. We found suggestive evidence (OR=1.001, 95% CI:1.000–1.001, *P*=0.0155) of genetic predictions of causality between schizophrenia and lung cancer; however, reverse causality could not be determined. This study demonstrated that schizophrenia is associated with an increased risk of lung cancer. A literature search revealed that the association between schizophrenia and lung cancer involves different mechanisms. Shaw et al. (44) showed that antipsychotic drugs fluspirilene, penfluridol, and pimozide induce apoptosis and inhibit metastasis through p53, STAT3, STAT5, protein phosphatase 2A, cholesterol homeostasis, integrin, autophagy, USP1, wnt/β-catenin signal transduction and DNA repair *in vivo* and *in vitro*. In addition, penfluridol and pimozide have synergistic effects with existing chemotherapeutic drugs such as cisplatin. Accumulating evidence suggests that antipsychotics are potential anticancer agents. Zuber et al. have shown that the relationship between schizophrenia and lung cancer may be related to common genetic risk factors; smoking is closely related to schizophrenia and lung cancer, and the variation in nicotinic acetylcholine receptors may lead to this overlap (43). In addition, the anti-schizophrenic drug penfluridol can inhibit the growth and metastasis of various G0/G1 stagnant lung cancer cells by increasing the expression of p21/p27 and reducing the expression of the cyclin-CDK complex. Penfluridol is an inexpensive drug approved by the Food and Drug Administration. This study demonstrated the potential of anti-schizophrenic drugs in the treatment of lung cancer.

This study had several advantages. First, we used two-sample MR analysis to minimize the deviation caused by confounding factors. Second, we performed an MR analysis of schizophrenia and 16 types of cancer; these 16 types of tumors are common and representative in the clinic. In addition, the database sources for each pair of exposures and results did not overlap and were of European origin, ensuring the basic requirements for the MR analysis of the two samples. Fourth, the exposed F-statistics were all greater than 10, indicating that there was no weak tool deviation. Fifth, to ensure the validity of the causal effect results, we conducted a sensitivity analysis, including the leave-one-out sensitivity, heterogeneity, and pleiotropy tests.

Despite these advantages, our study had certain limitations. First, we used only GWAS data on PGC schizophrenia; therefore, the results may be inadequate. In addition, because the two samples of the MR analysis required ethnic consistency, our study focused on the European population and did not cover the Asian and African populations, which may have led to incomplete results. In addition, population stratification may interfere with the causal relationship between schizophrenia and tumors (45). Although the population studied was European, the internal structure of the population and potential sex factors were not considered. Sex differences in the incidence of schizophrenia have been reported (46), and sex differences between schizophrenia and lung cancer have gained considerable attention. Furthermore, on reversing the MR analysis, we found that the number of strong correlations SNP was relatively small, resulting in invalid results that limited our ability to draw real causality conclusions. In addition, the OR value is 1.001, indicating that there is a potential correlation between schizophrenia and lung cancer. Schizophrenia is a risk factor for lung cancer, but the possible risk factors are not very obvious. It is necessary to further explore the relationship between schizophrenia and lung cancer in the future. This result also suggests that we need to pay more attention to the incidence of lung cancer in patients with schizophrenia in the future. Finally, our study aimed to determine whether there is a causal relationship between schizophrenia and lung cancer; however, the underlying mechanism has not yet been studied. Future investigations analyzing the possible mechanisms increasing the risk of lung cancer in patients with schizophrenia are warranted.

## 5 Conclusion

A two-sample MR analysis revealed a potential causal relationship between the genetic prediction of schizophrenia and lung cancer using schizophrenia as an exposure factor and 16 cancer types as outcomes. However, the causal relationship between lung cancer and schizophrenia has not been determined, and the causal effect and reverse causal relationship of schizophrenia on other types of cancer have not been determined. This demonstrates the importance of preventing schizophrenia in the prevention and treatment of lung cancer. Future large-scale prospective studies and more in-depth mechanism research are required to validate the study findings.

## Data availability statement

The datasets presented in this study can be found in online repositories. The names of the repository/repositories and accession number(s) can be found in the article/**Supplementary Material**.

## Author contributions

XTZ: Writing – original draft, Writing – review & editing. QL: Writing – original draft, Writing – review & editing. SHL: Writing –review & editing. ZLS: Writing – review & editing. LQW: Writing – review & editing. CGS: Writing – review & editing. XNC: Writing – review & editing.
