# Supplementary material for: Genetic prediction of the causal relationship between schizophrenia and tumors: a Mendelian randomized study
Source: Front Oncol. 2024 Feb 16;14:1321445. doi: 10.3389/fonc.2024.1321445 (PMC10905381; doi:10.3389/fonc.2024.1321445)

**1.MR leave-one-out sensitivity analysis for Schizophrenia on Lung cancer**
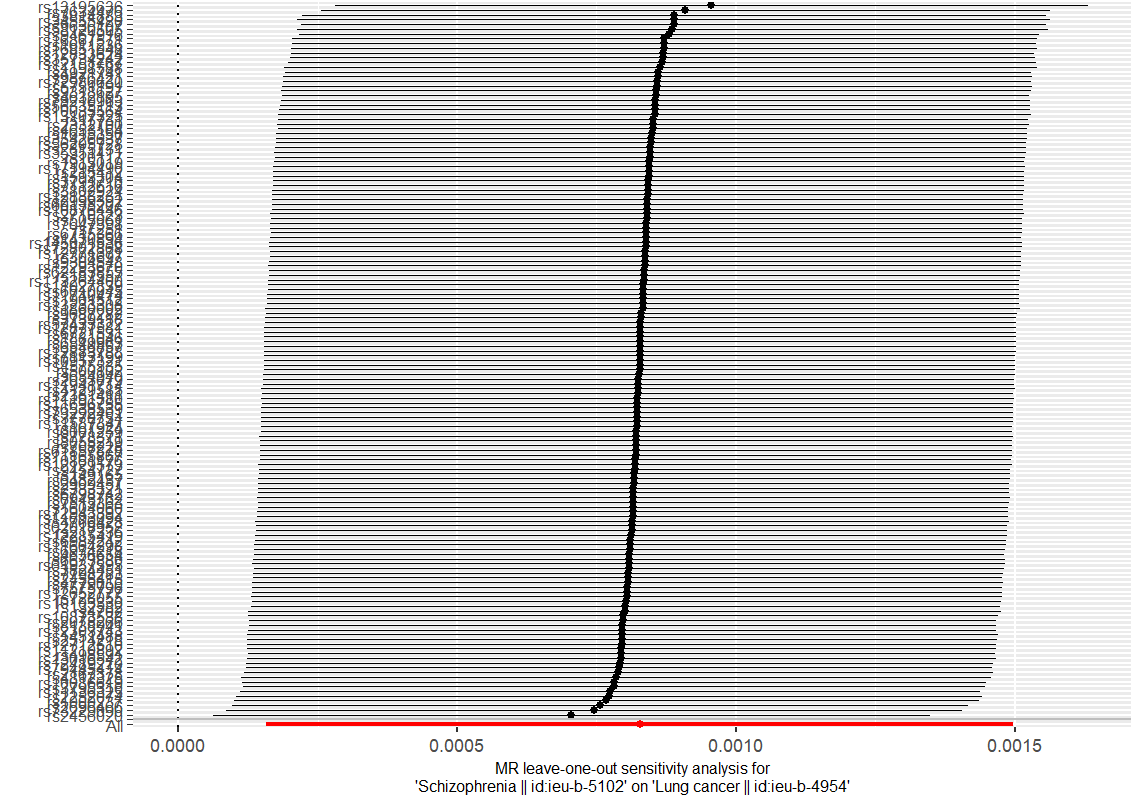


**MR Scatter plot**

**
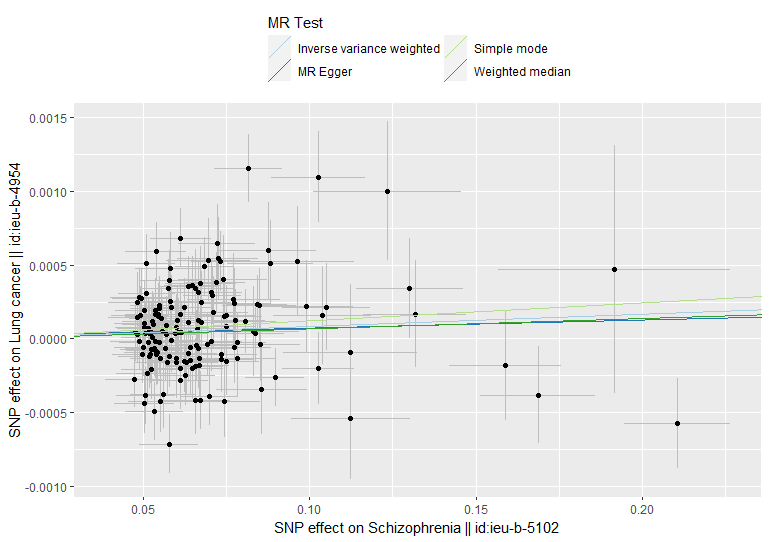
Forest map**


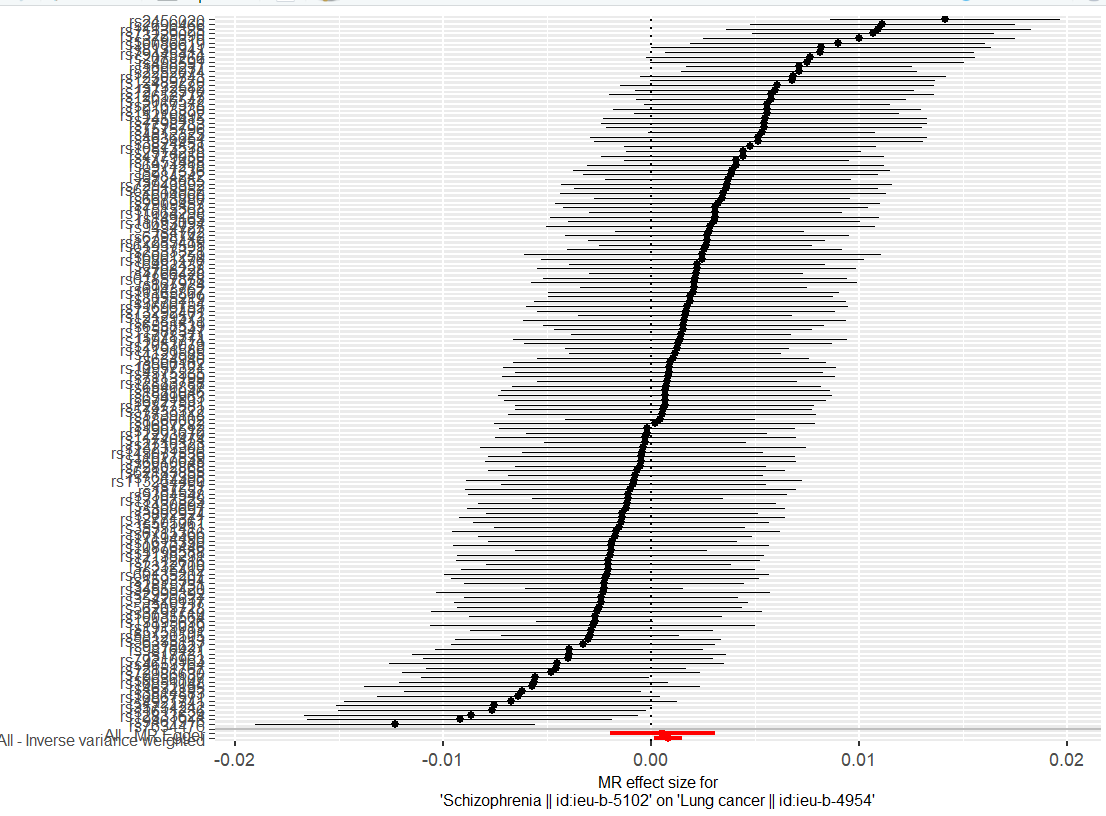


**Funnel plot**


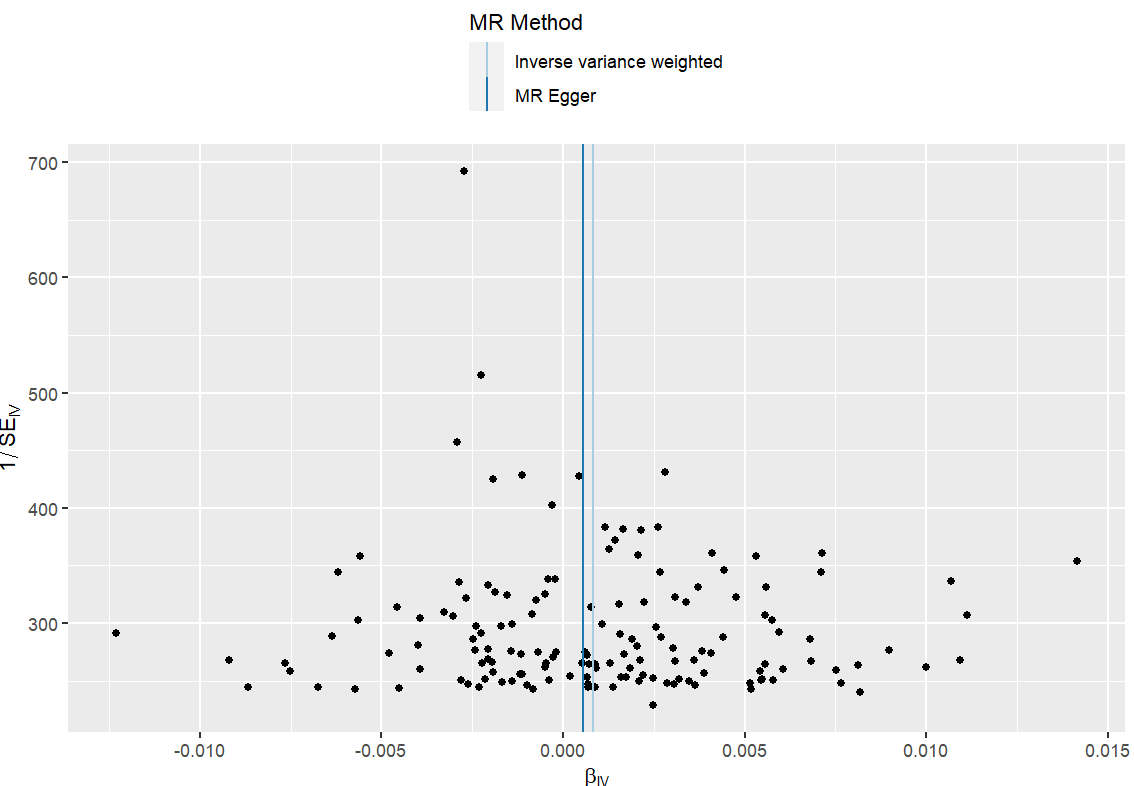


**2. MR leave-one-out sensitivity analysis for Schizophrenia on Adenocarcinoma of lung**
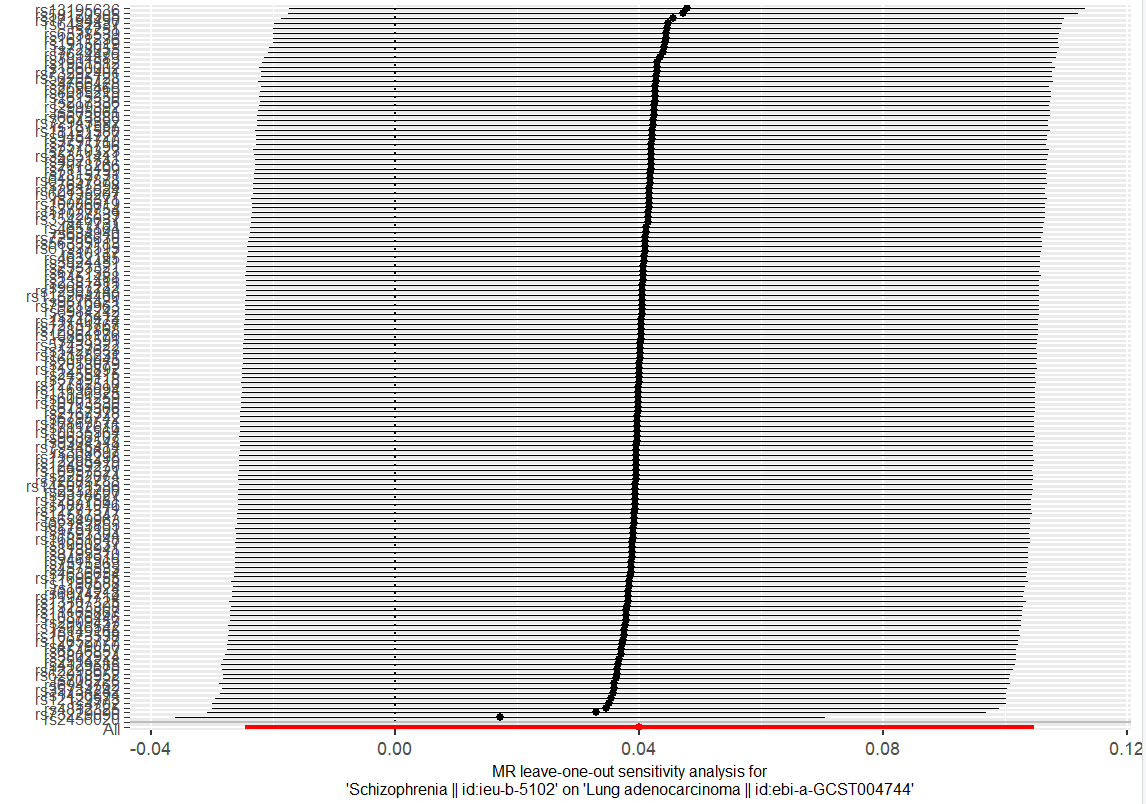


**MR Scatter plot**


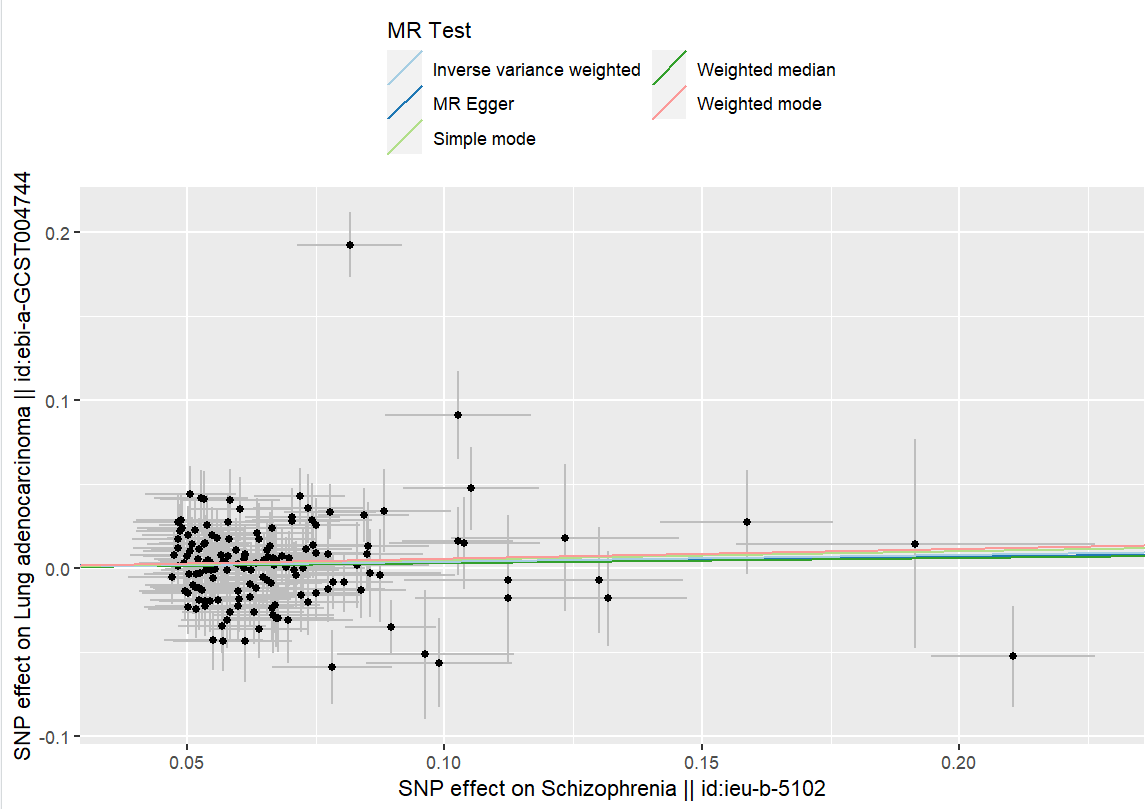


**Forest map**


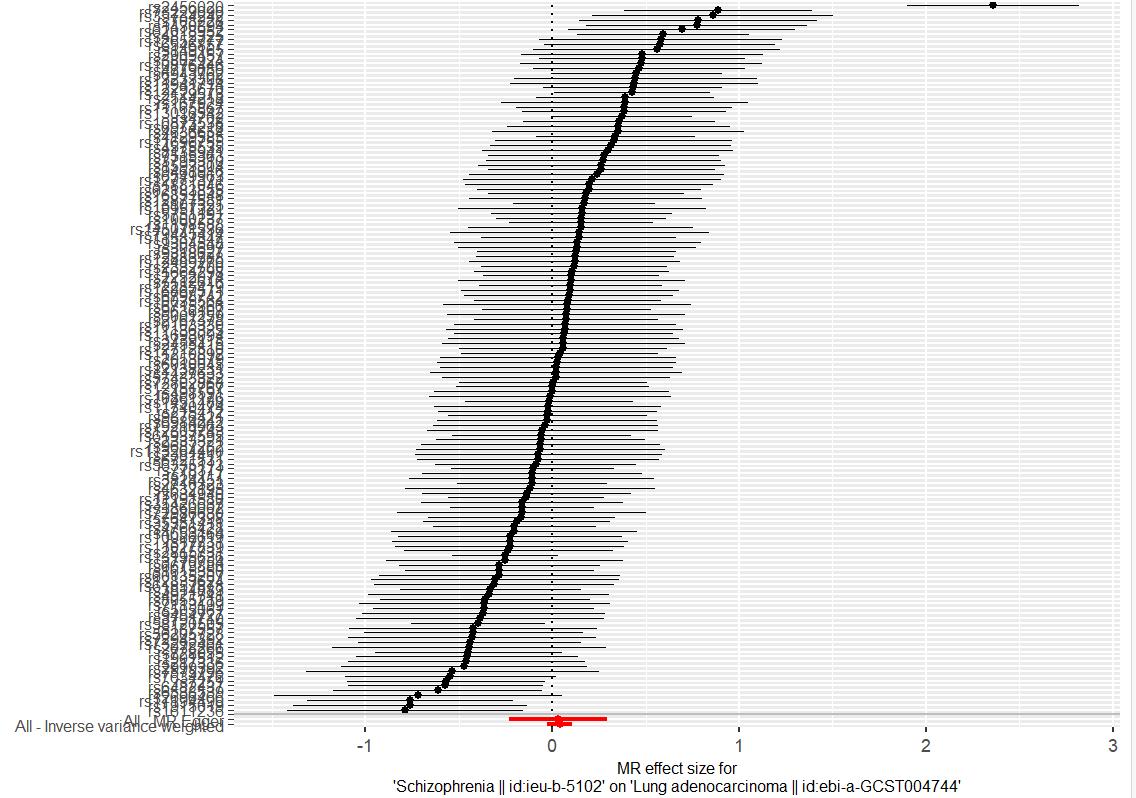


**Funnel plot**


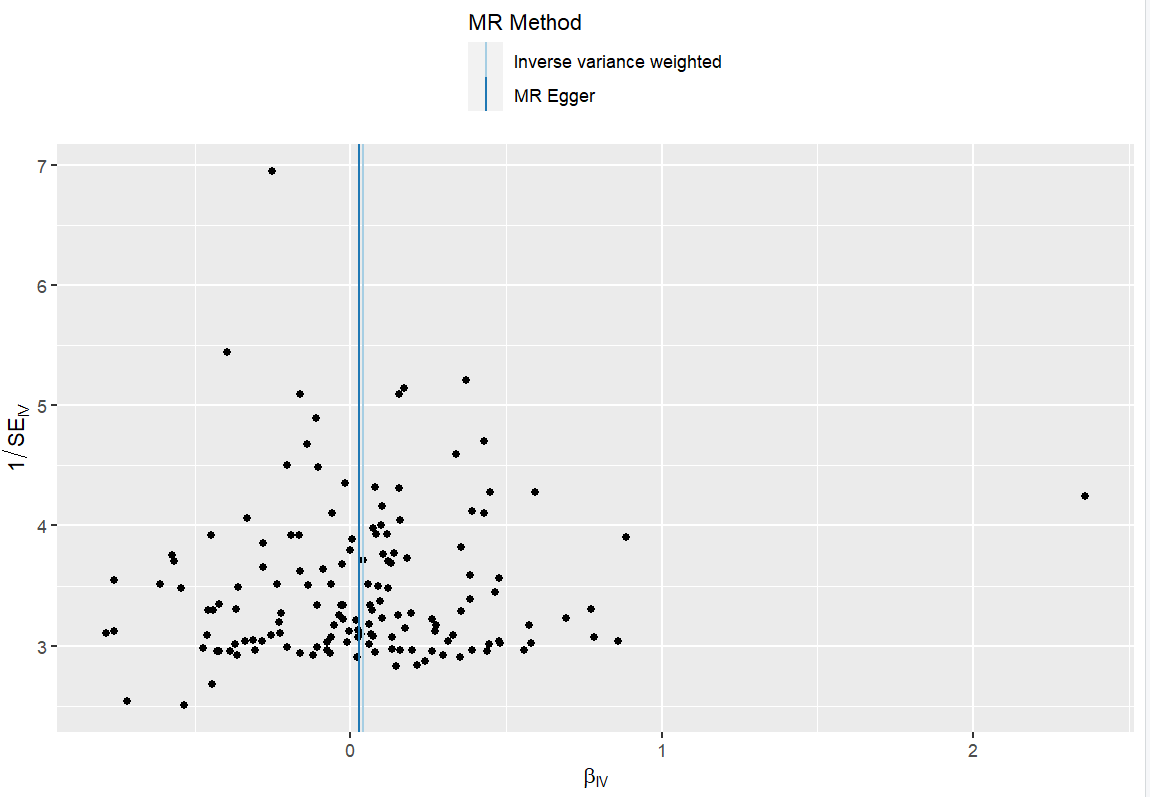


**3. MR leave-one-out sensitivity analysis for Schizophrenia on Squamous cell carcinoma of lung**
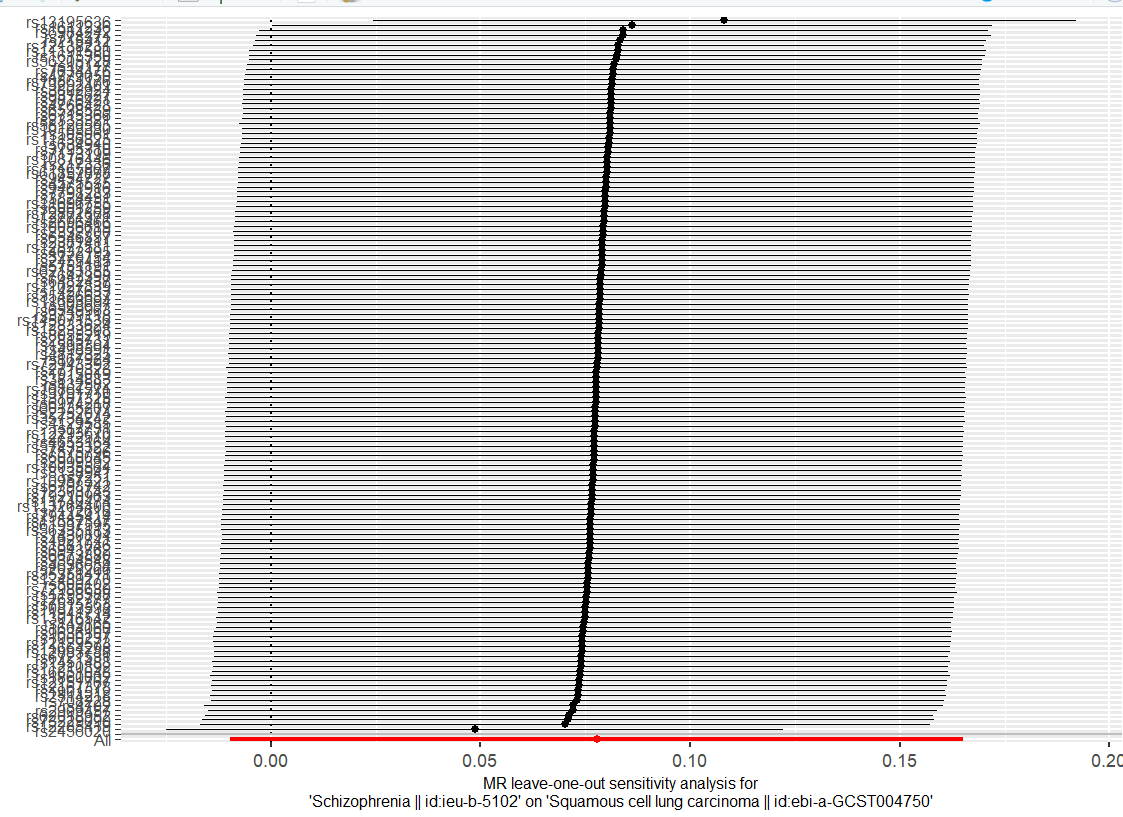


MR Scatter plot


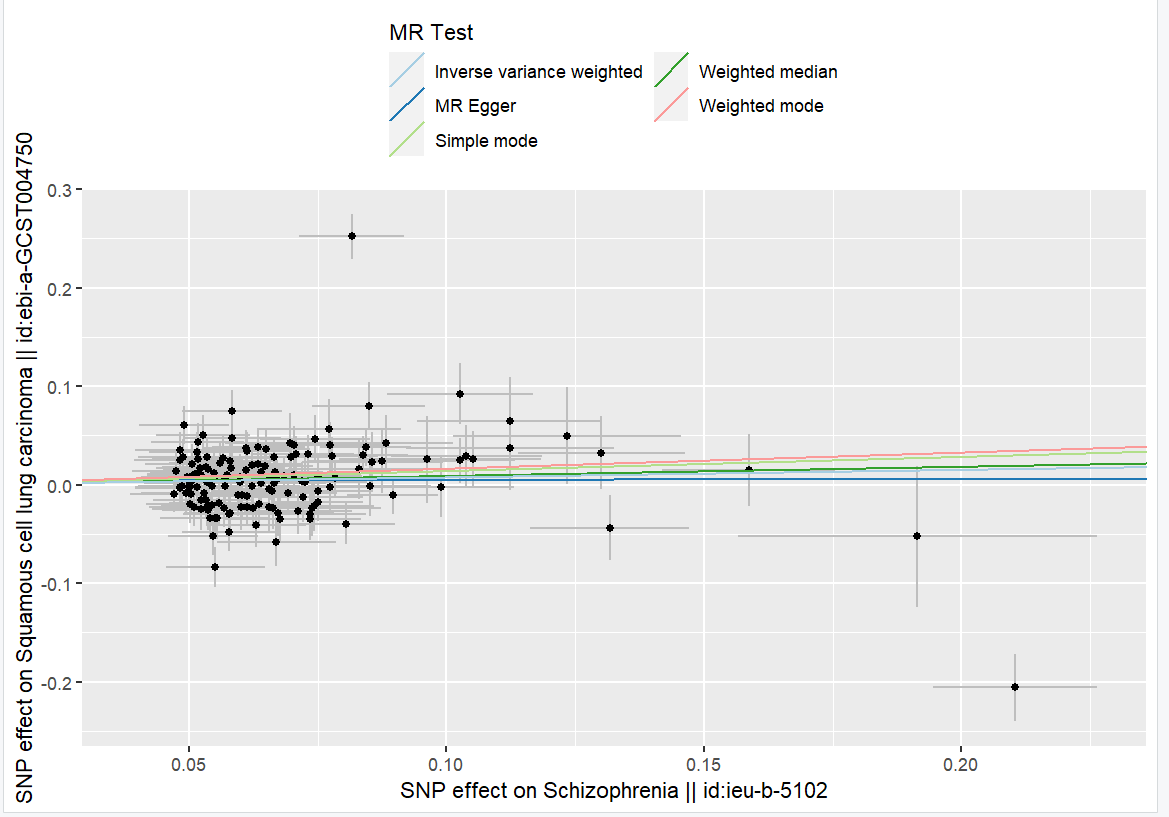


Forest map


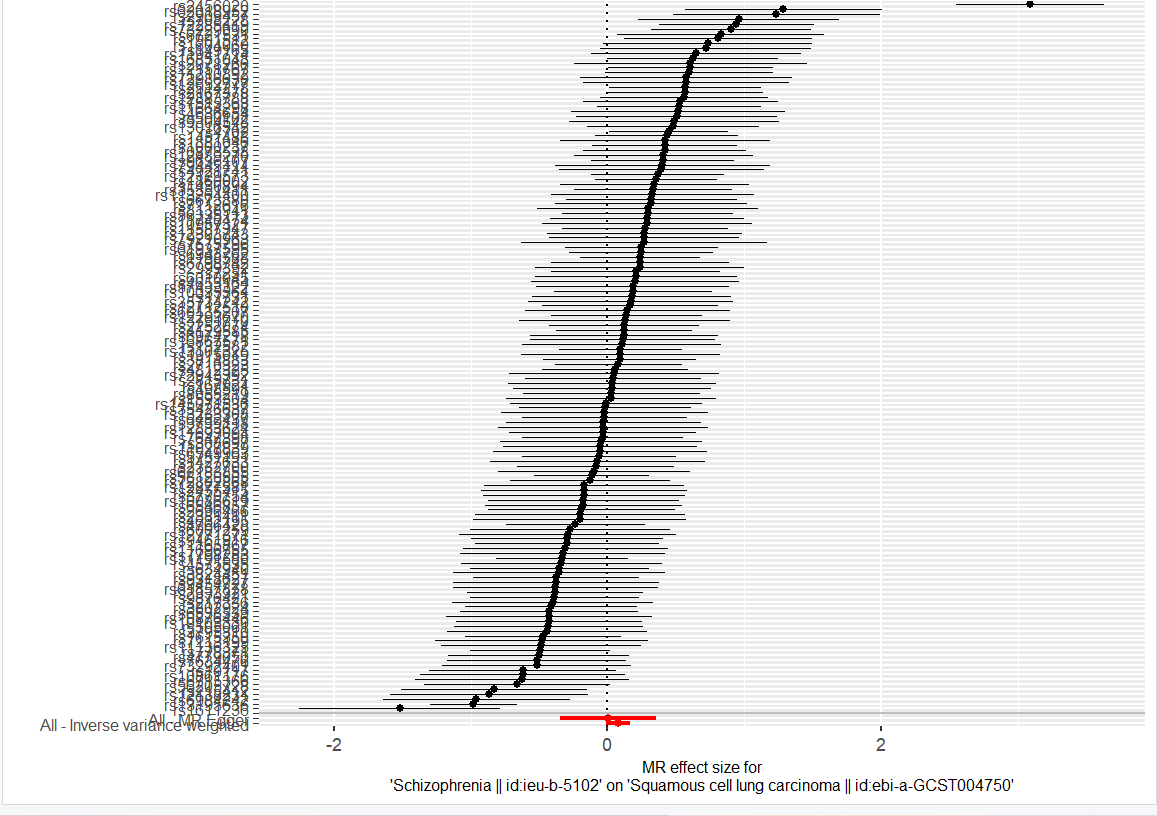


Funnel plot


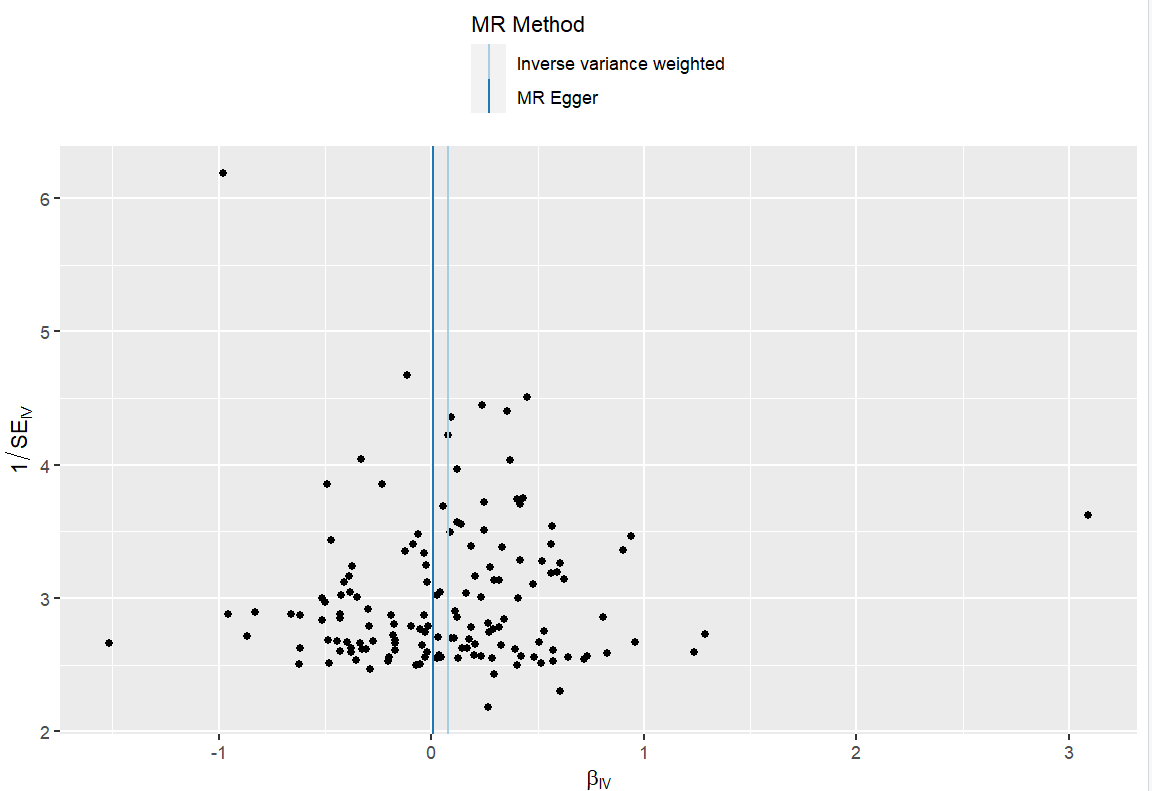


4. MR leave-one-out sensitivity analysis for Schizophrenia on Small cell lung cancer
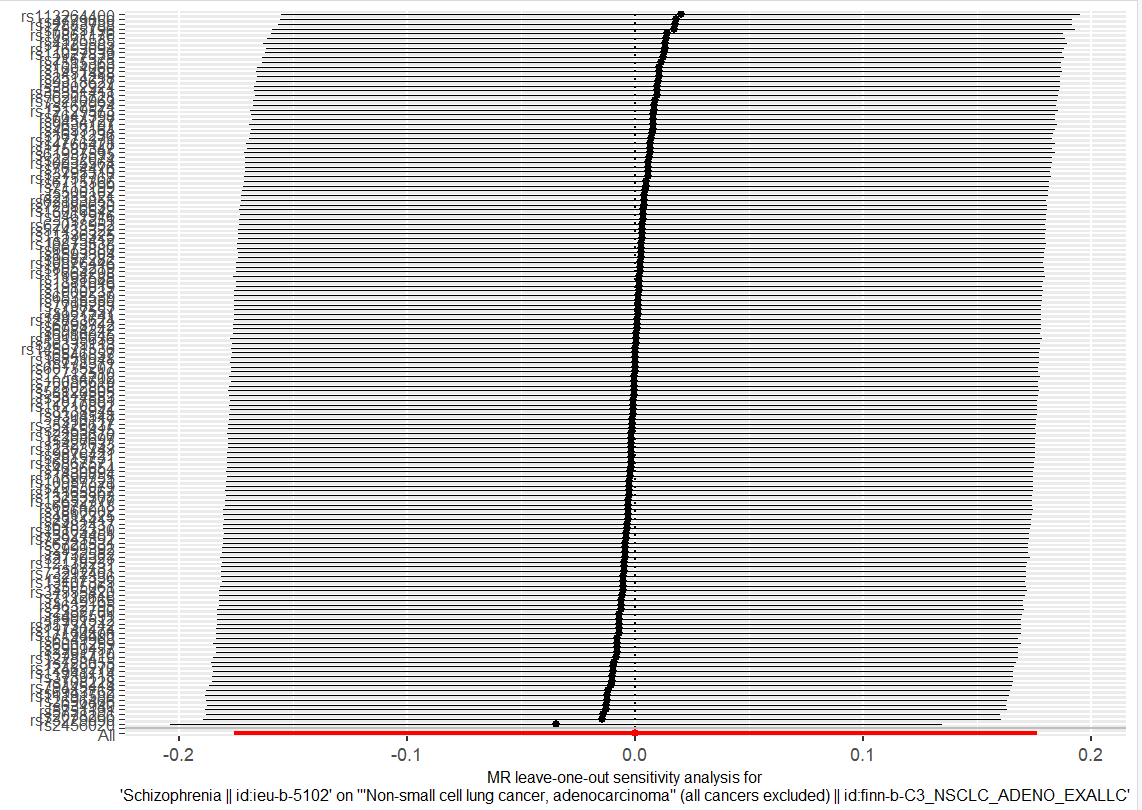


MR Scatter plot


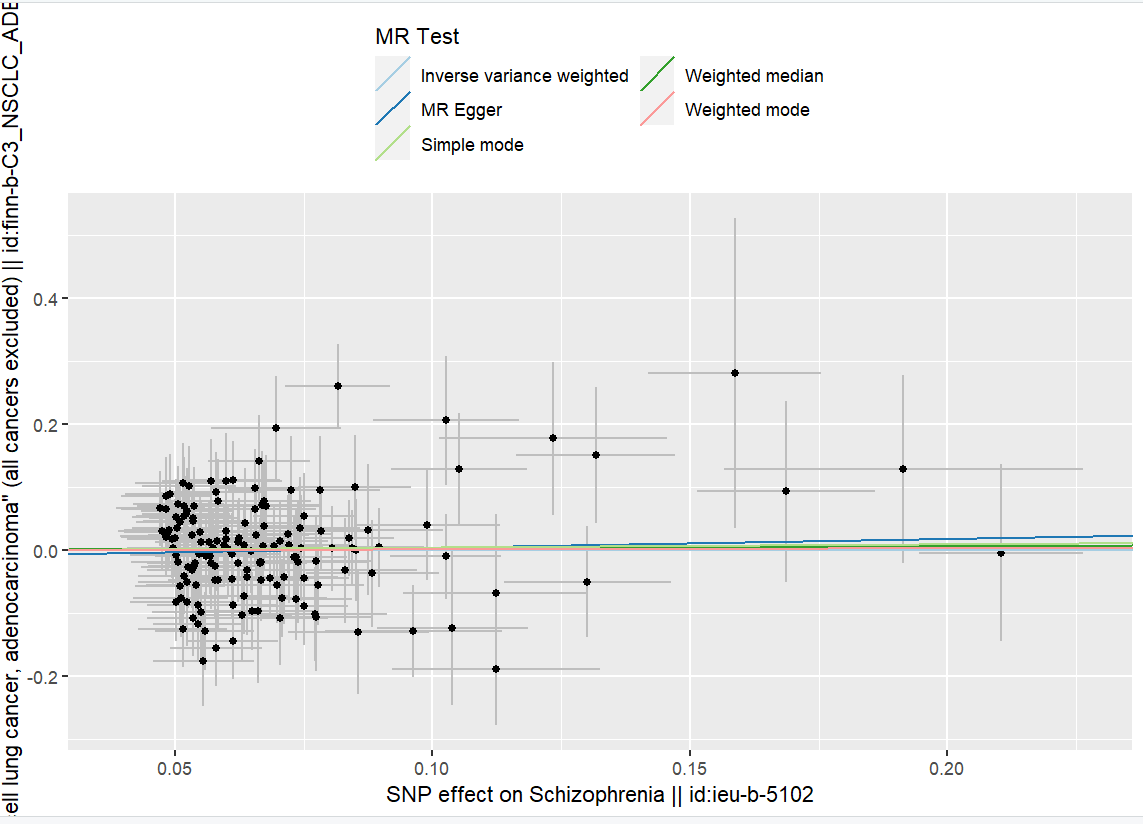


Forest map


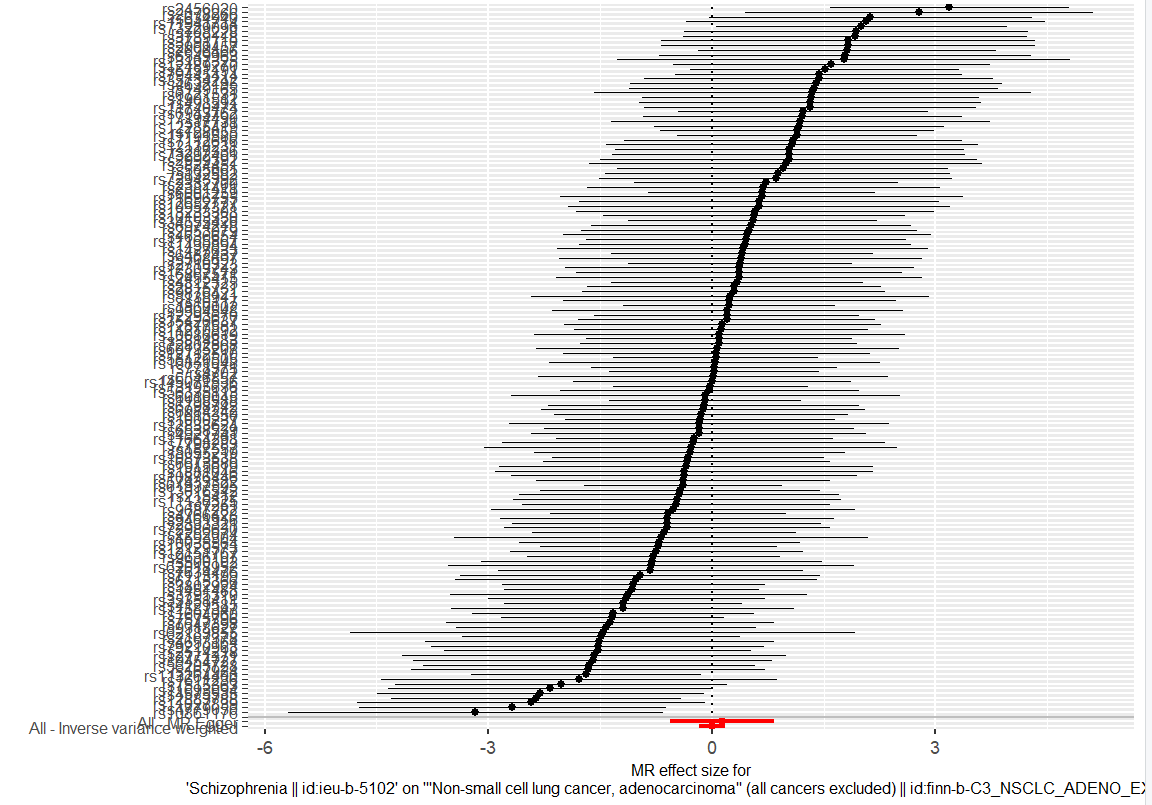


Funnel plot


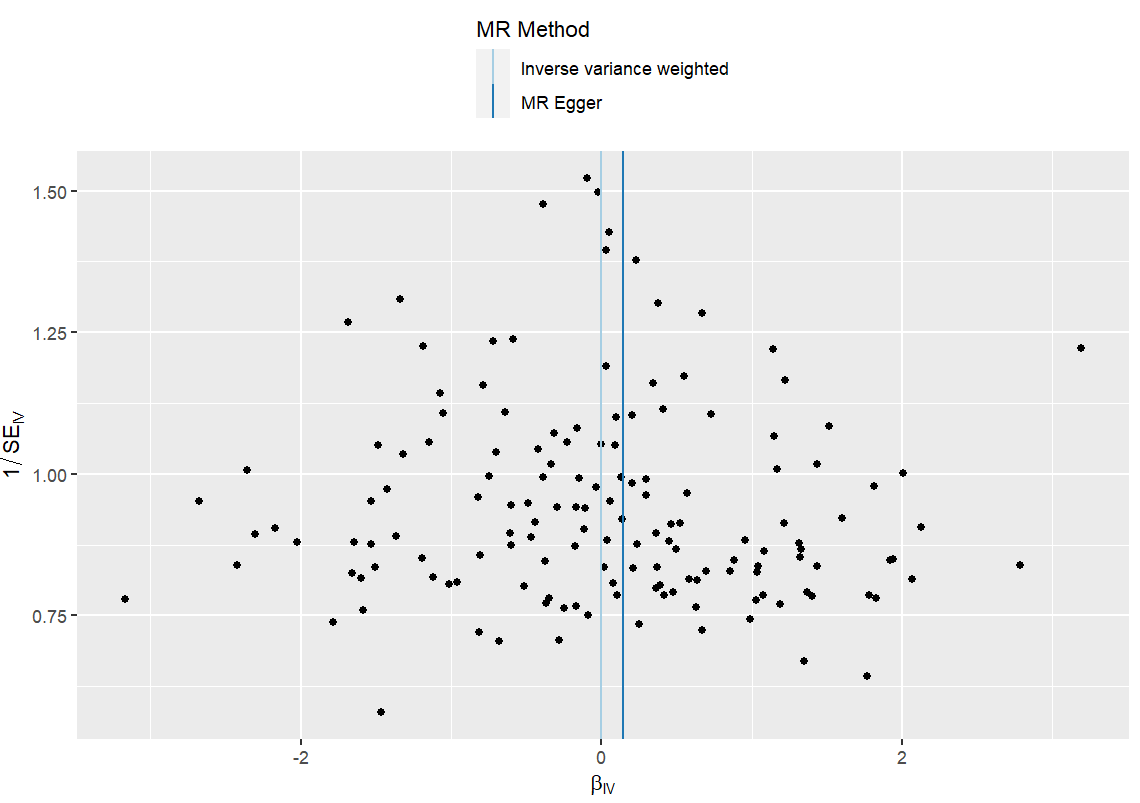


5.MR leave-one-out sensitivity analysis for Schizophrenia on breast cancer
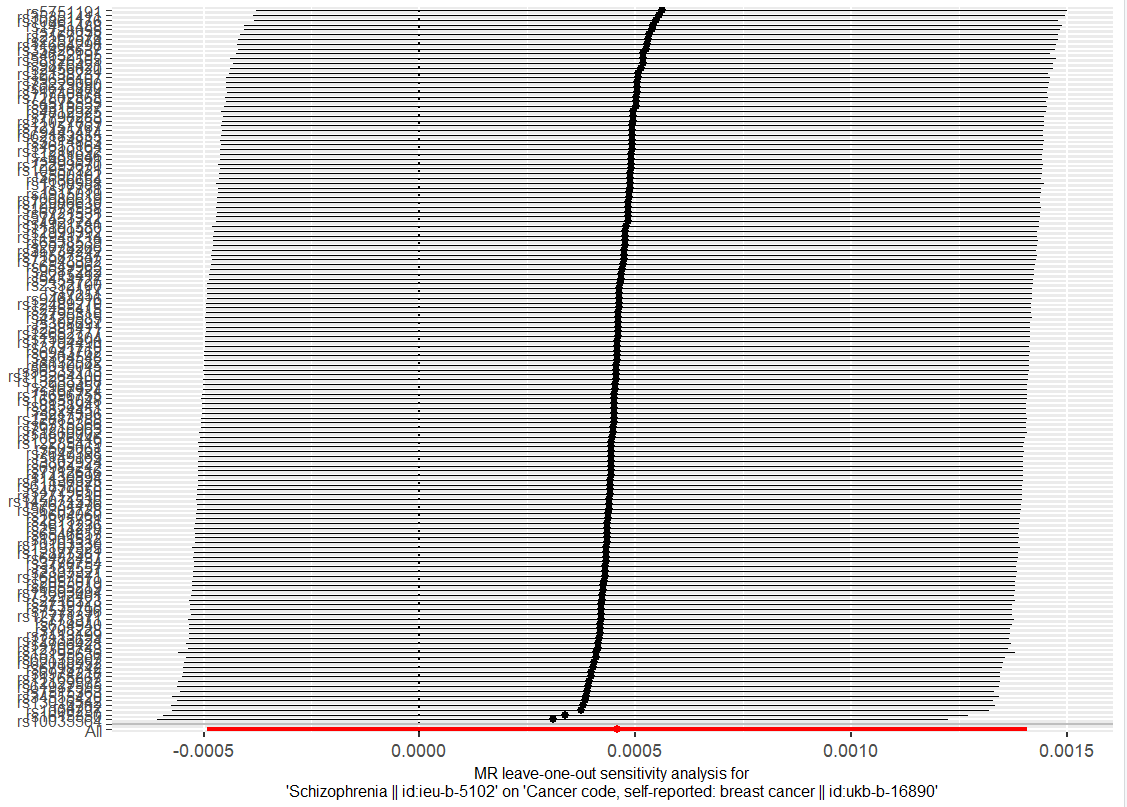


MR Scatter plot


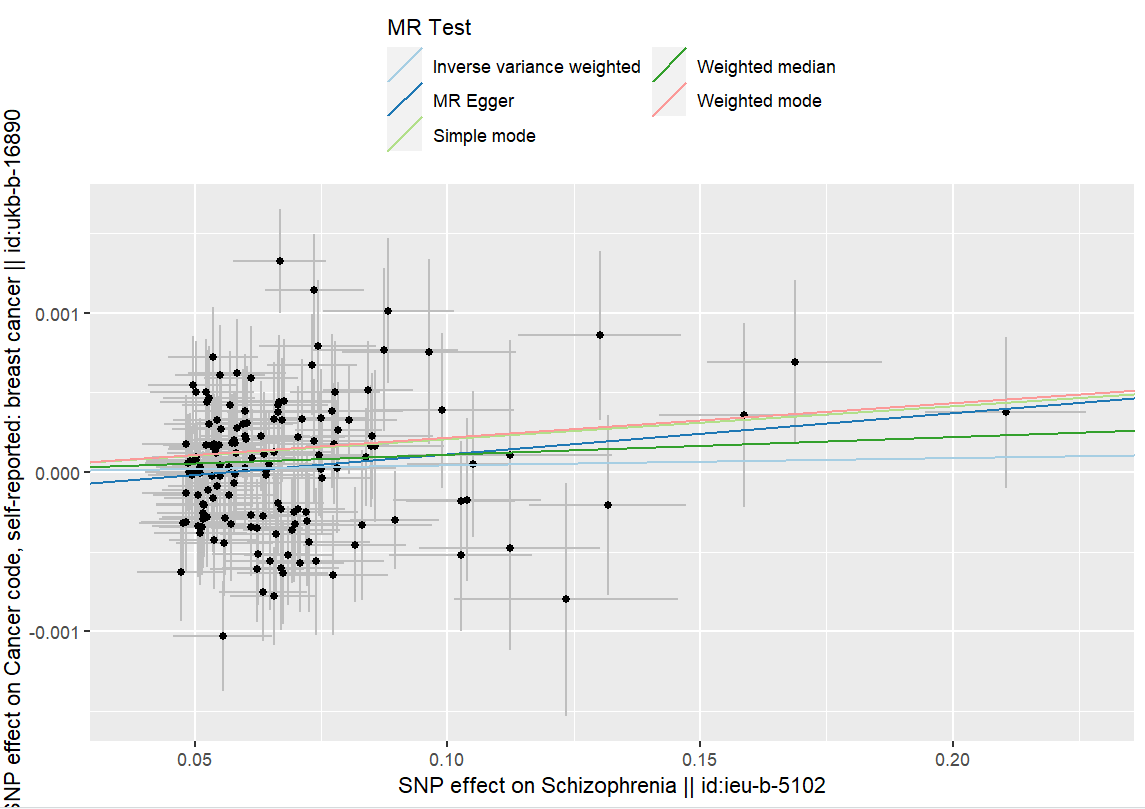


Forest map


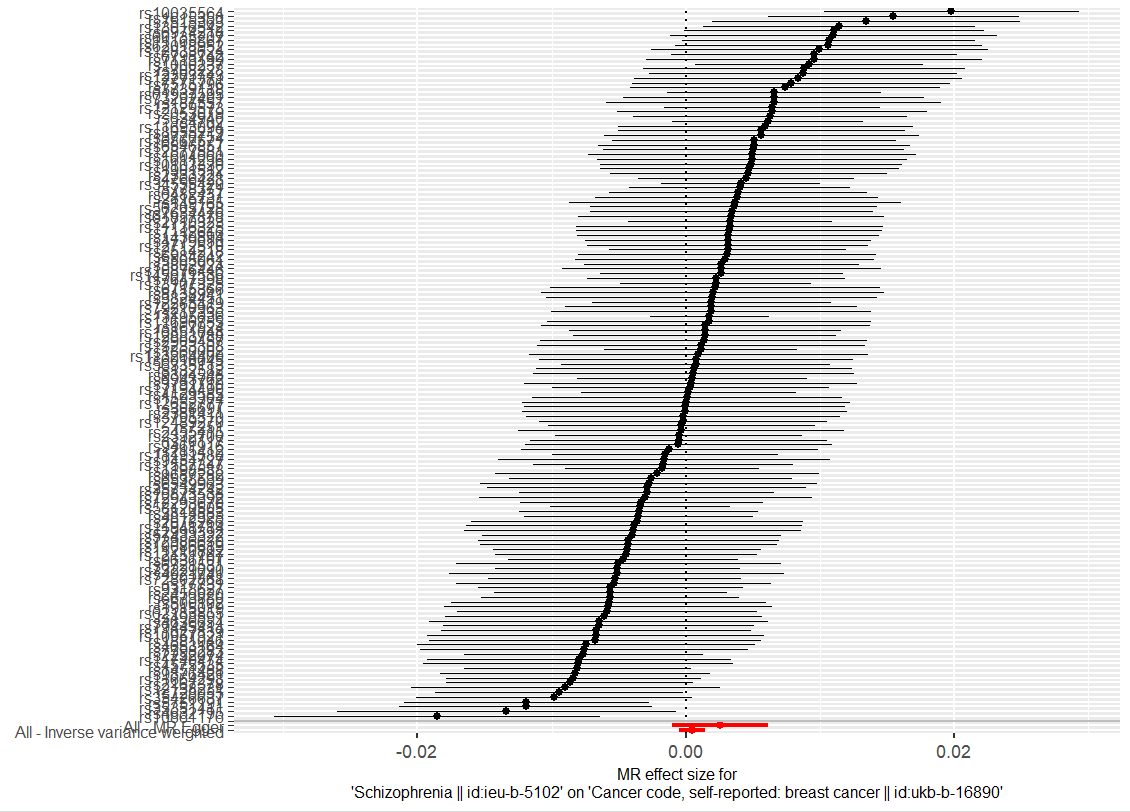


Funnel plot


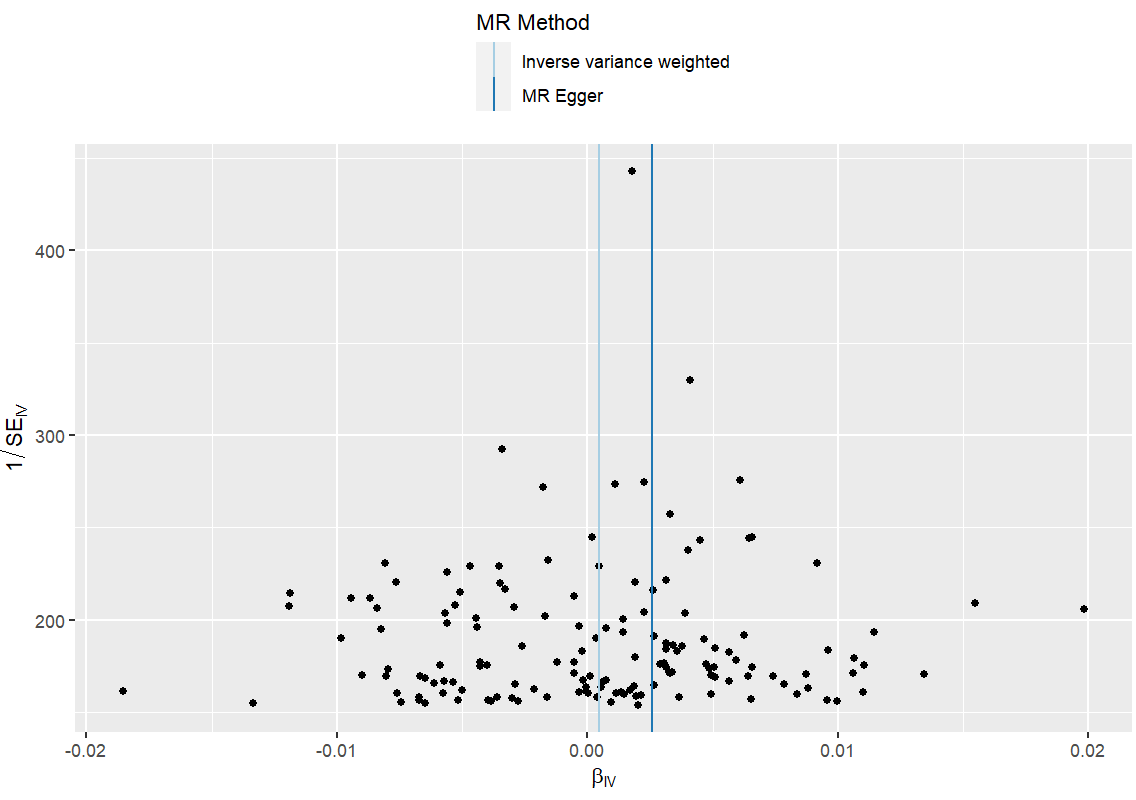


6. MR leave-one-out sensitivity analysis for Schizophrenia on Thyroid cancer
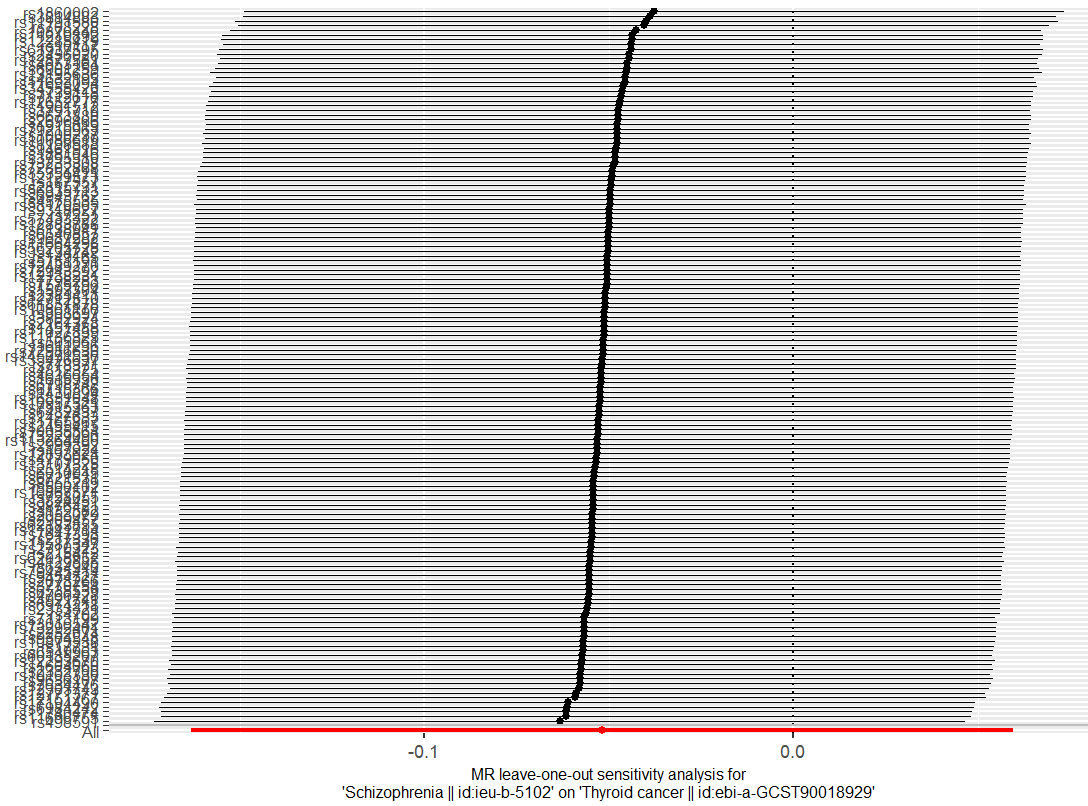


MR Scatter plot


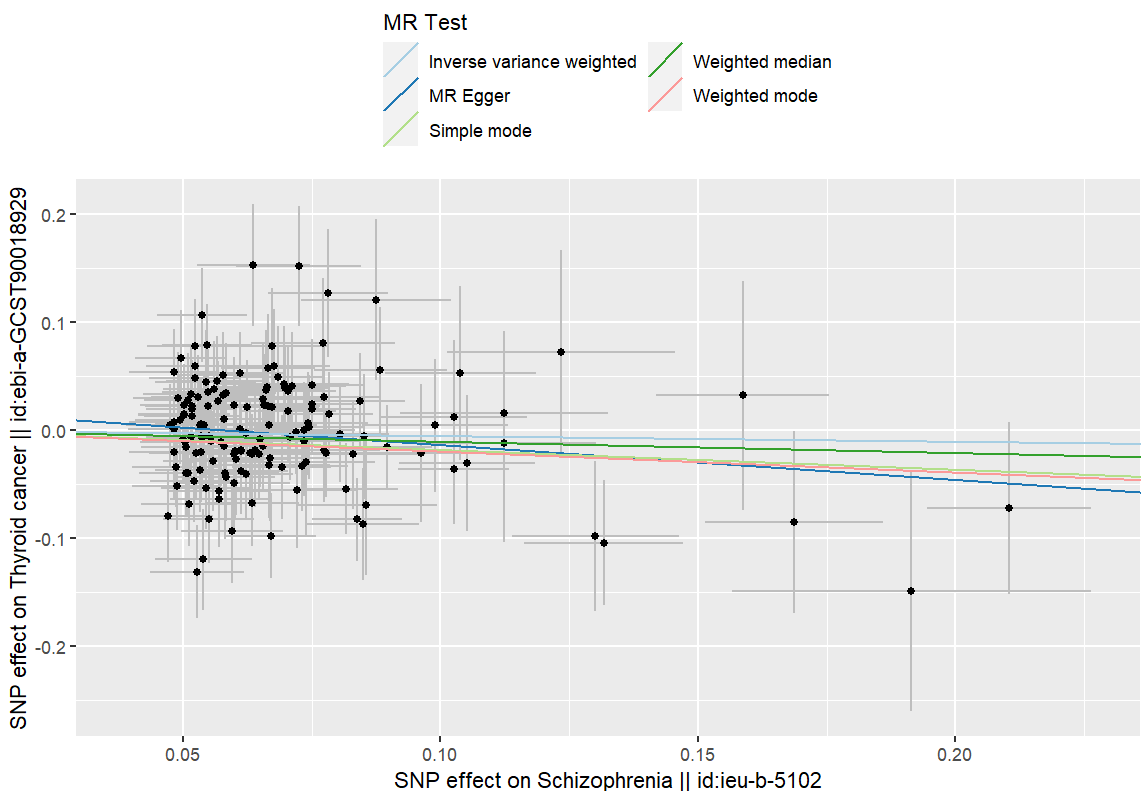


Forest map


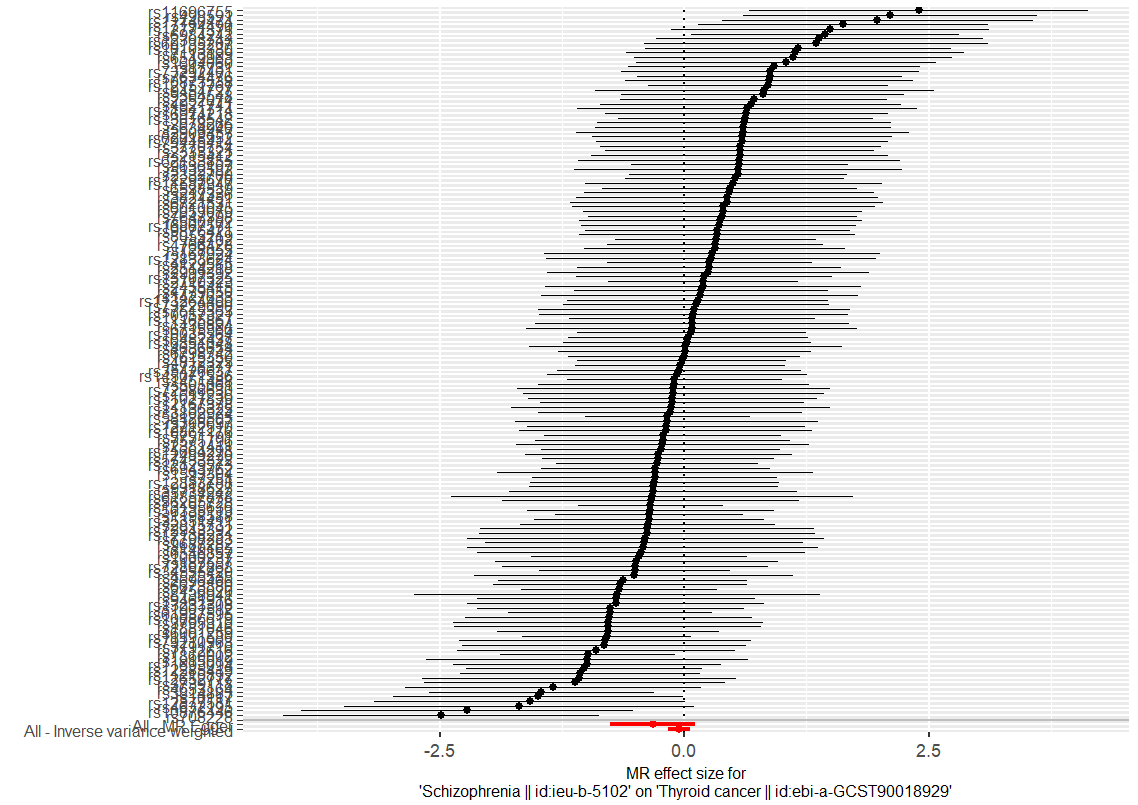


Funnel plot


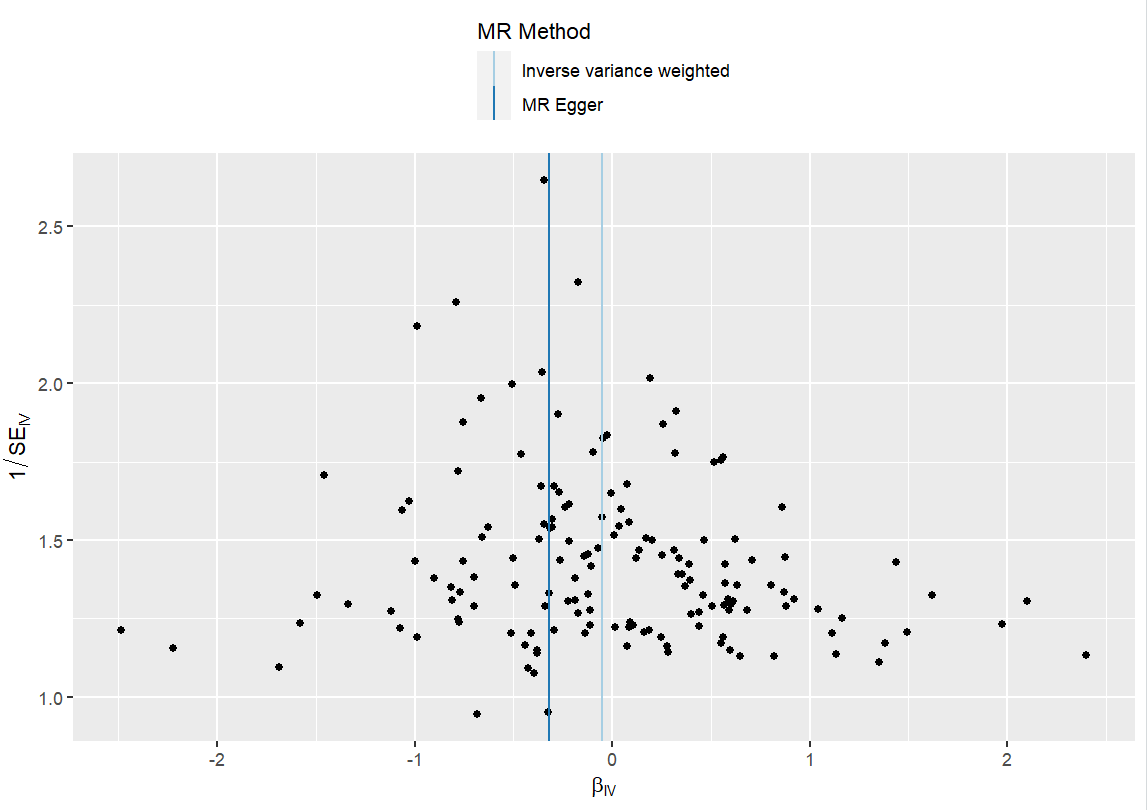


7.MR leave-one-out sensitivity analysis for Schizophrenia on Gastric cancer
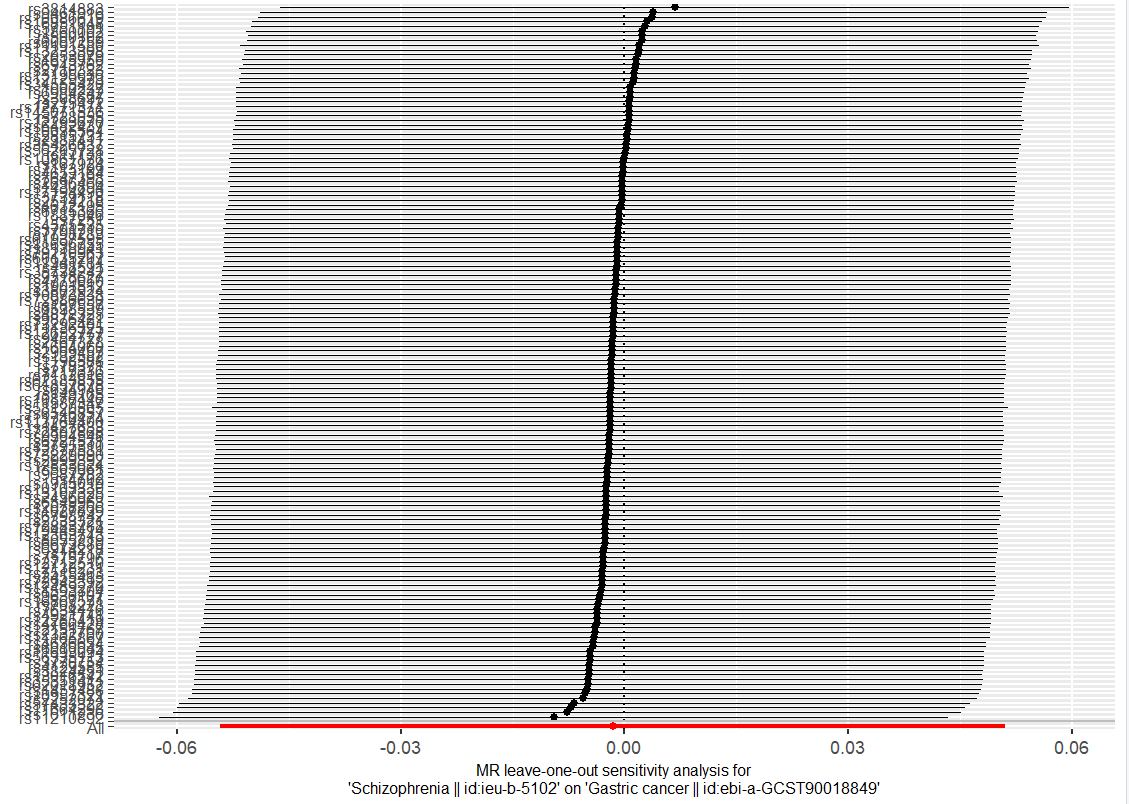


MR Scatter plot


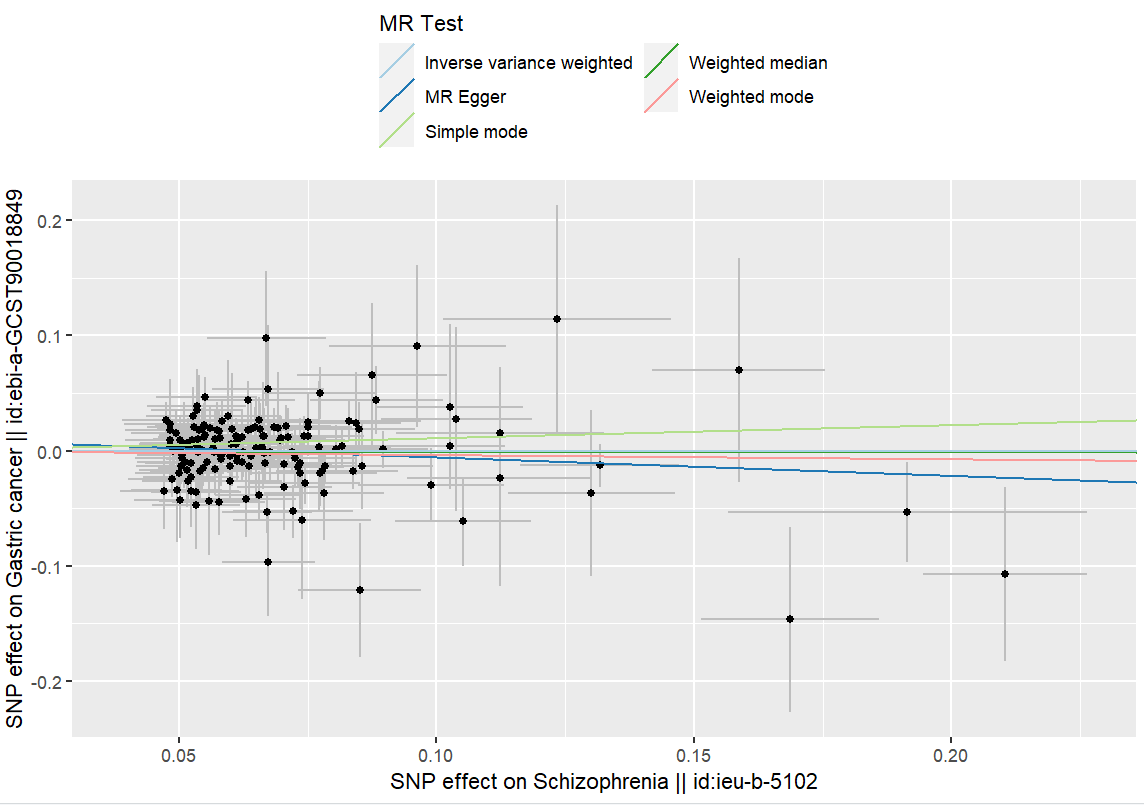


Forest map


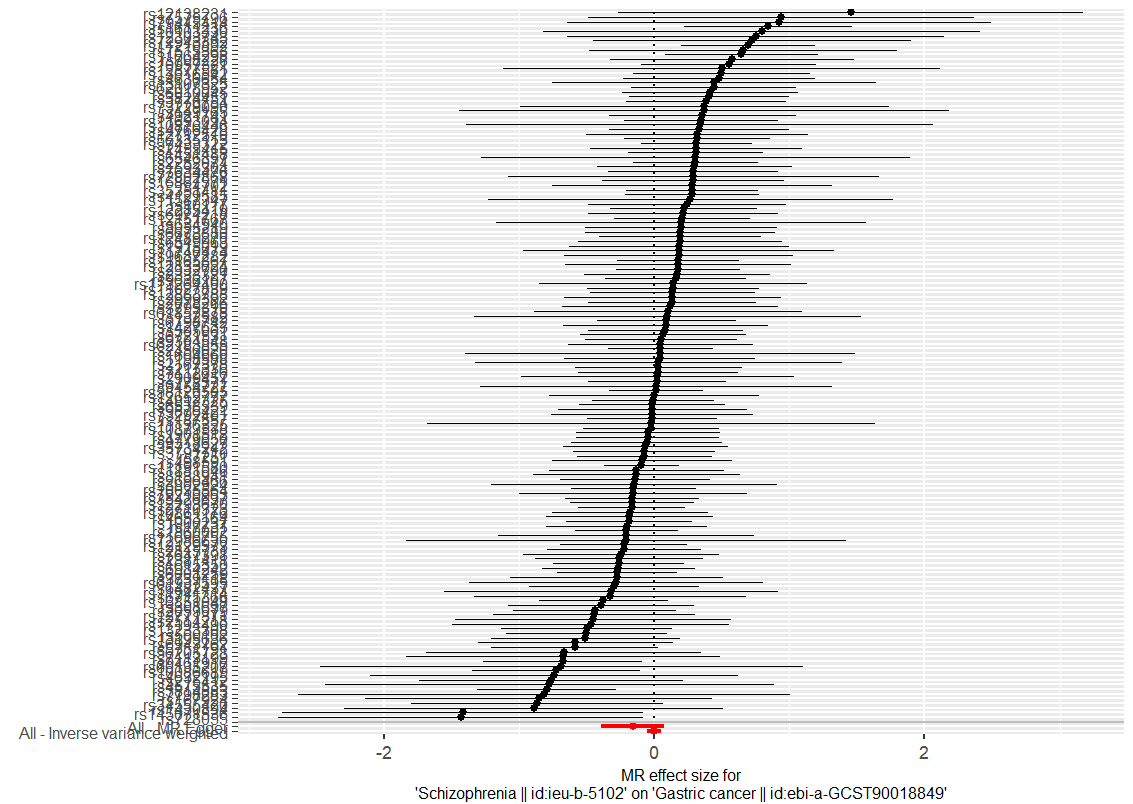


Funnel plot


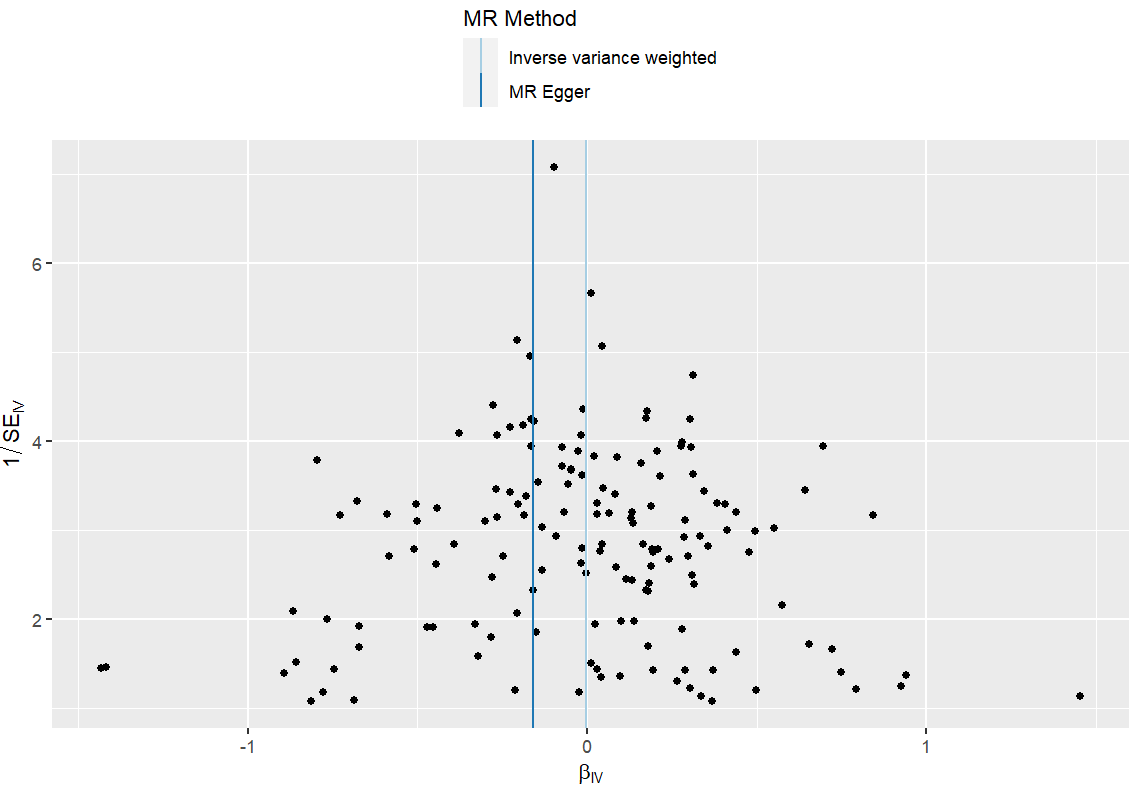


8. MR leave-one-out sensitivity analysis for Schizophrenia on Pancreatic cancer
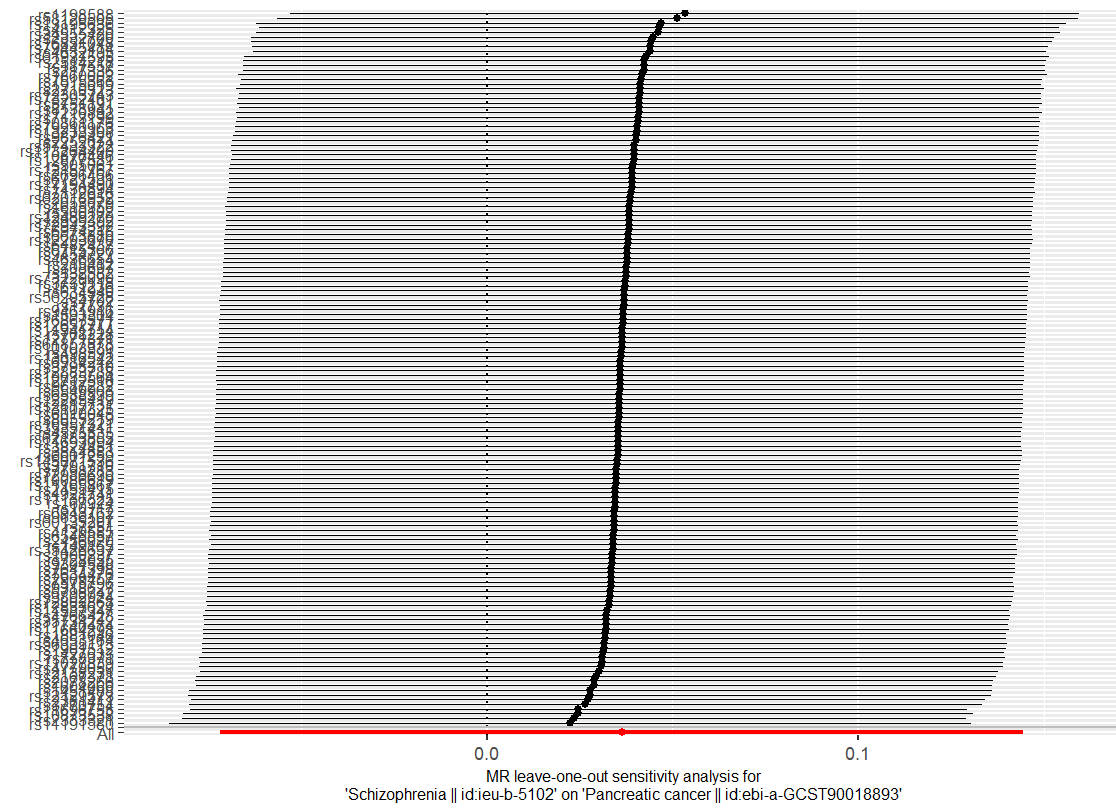


MR Scatter plot


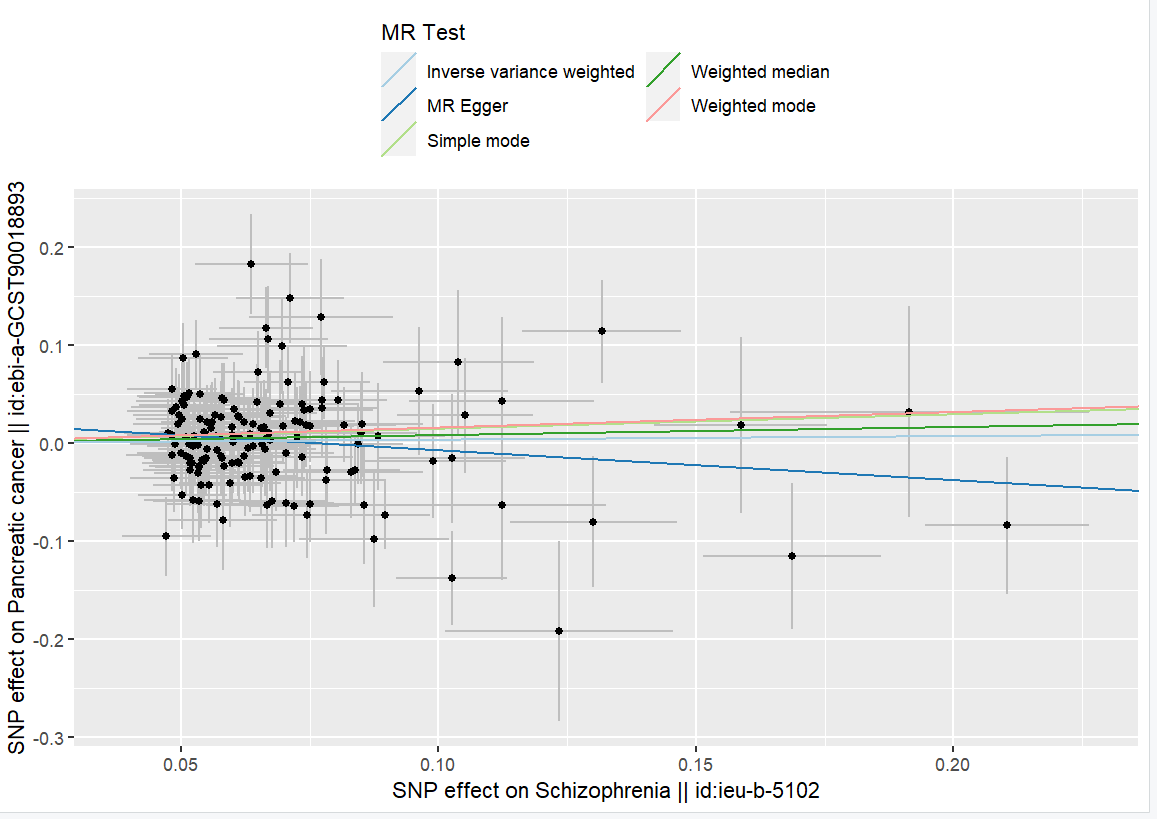


Forest map


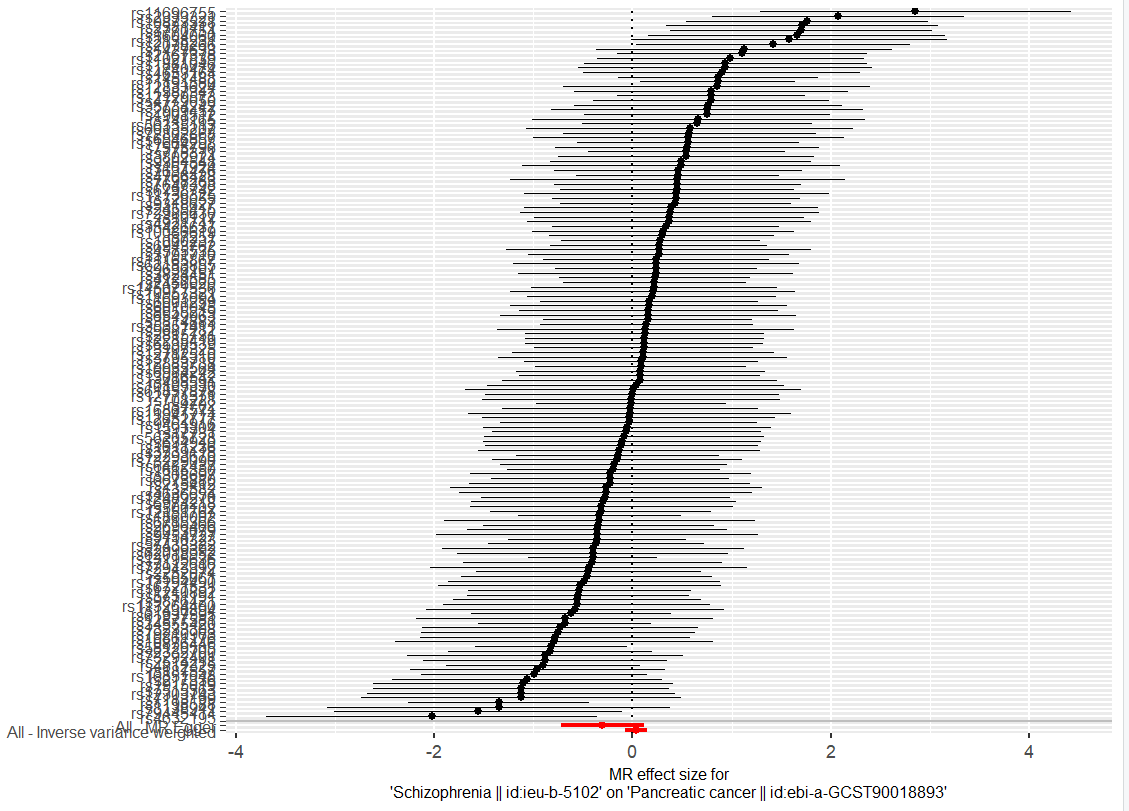


Funnel plot


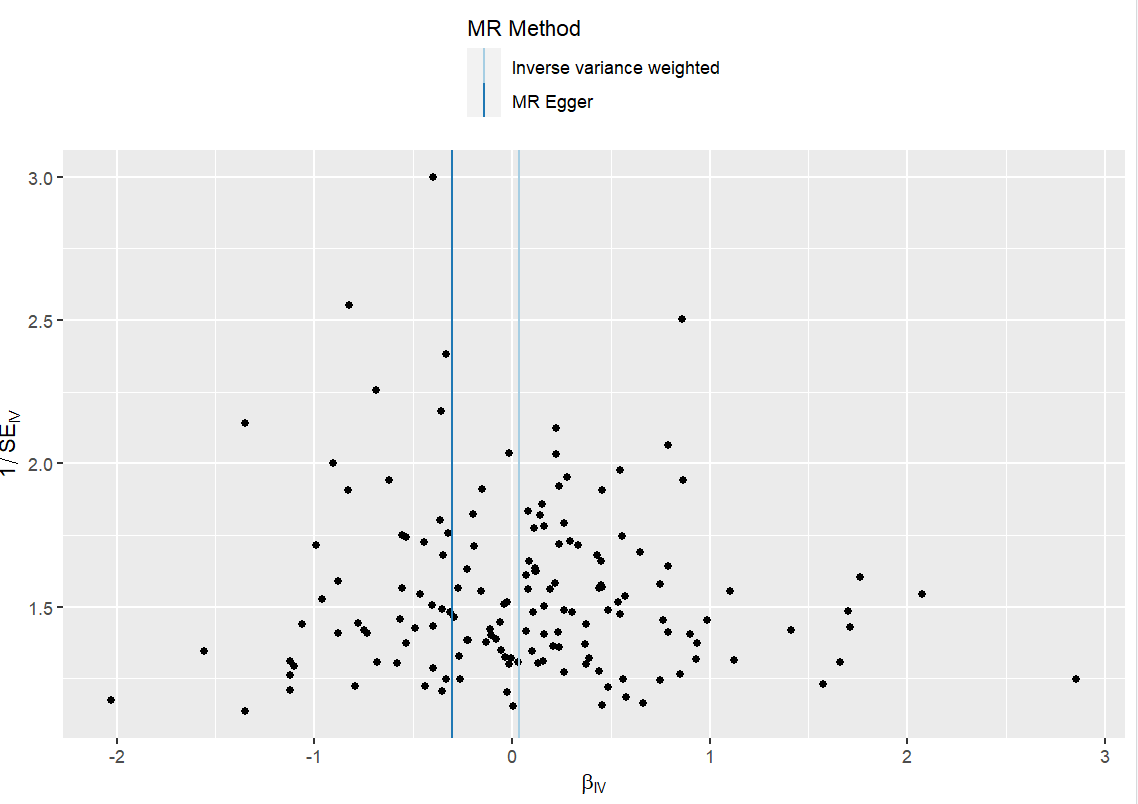


9. MR leave-one-out sensitivity analysis for Schizophrenia on Alcohol-related hepatocellular carcinoma
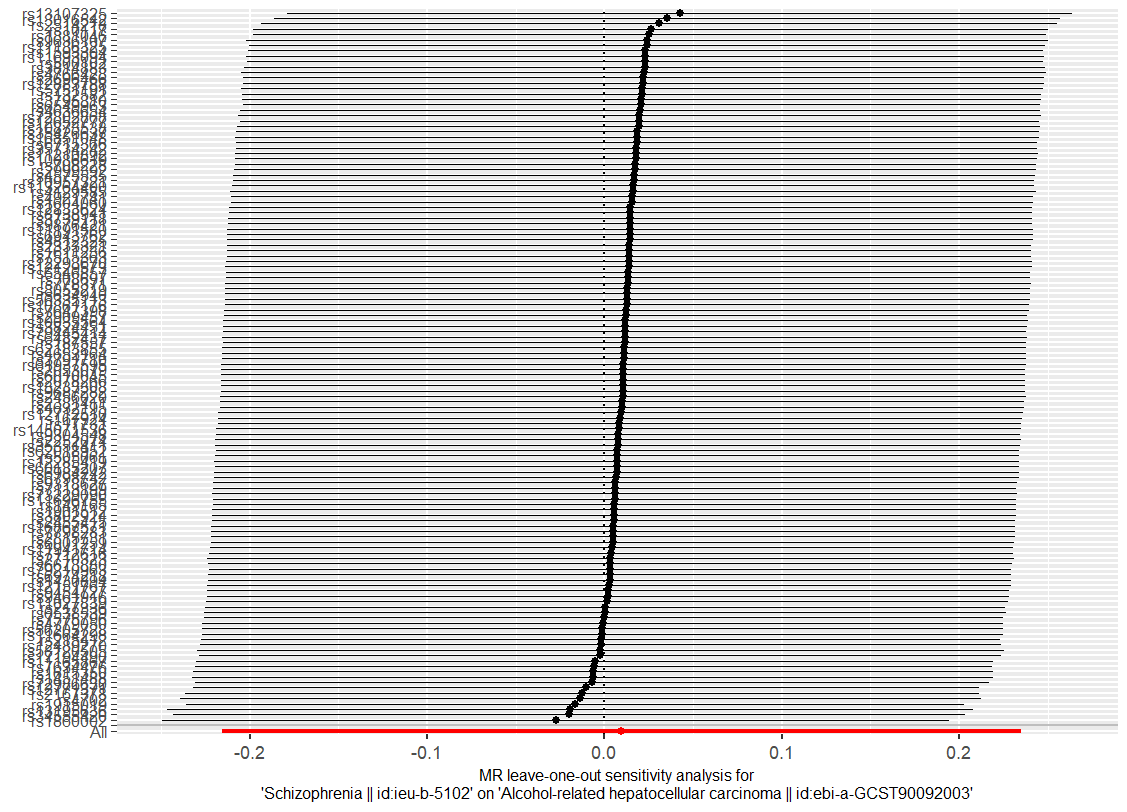


MR Scatter plot


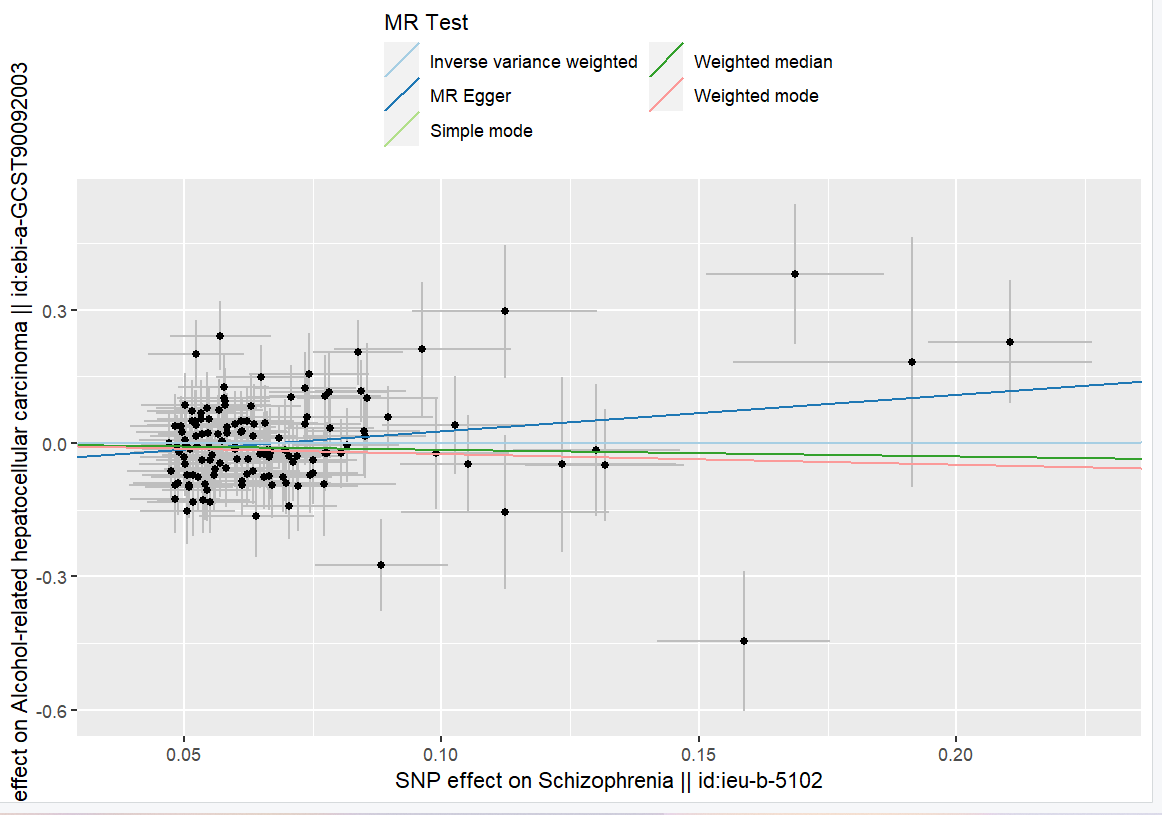


Forest map


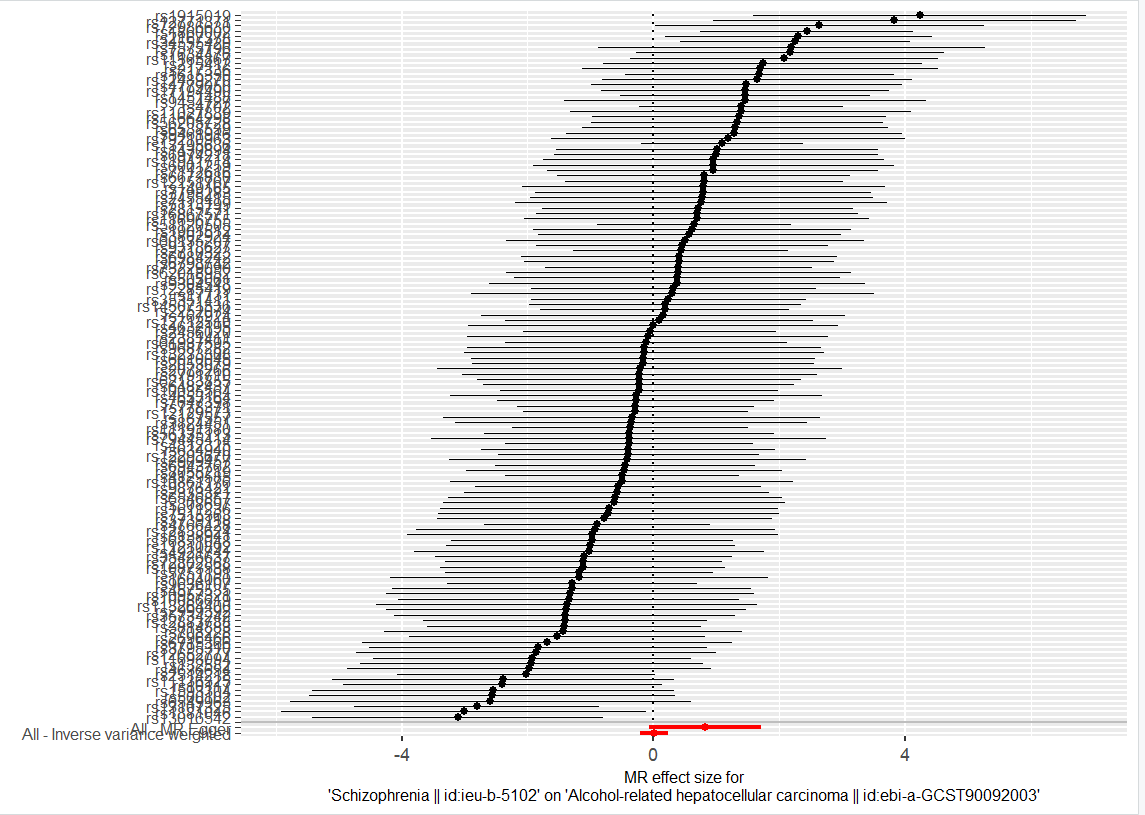


Funnel plot


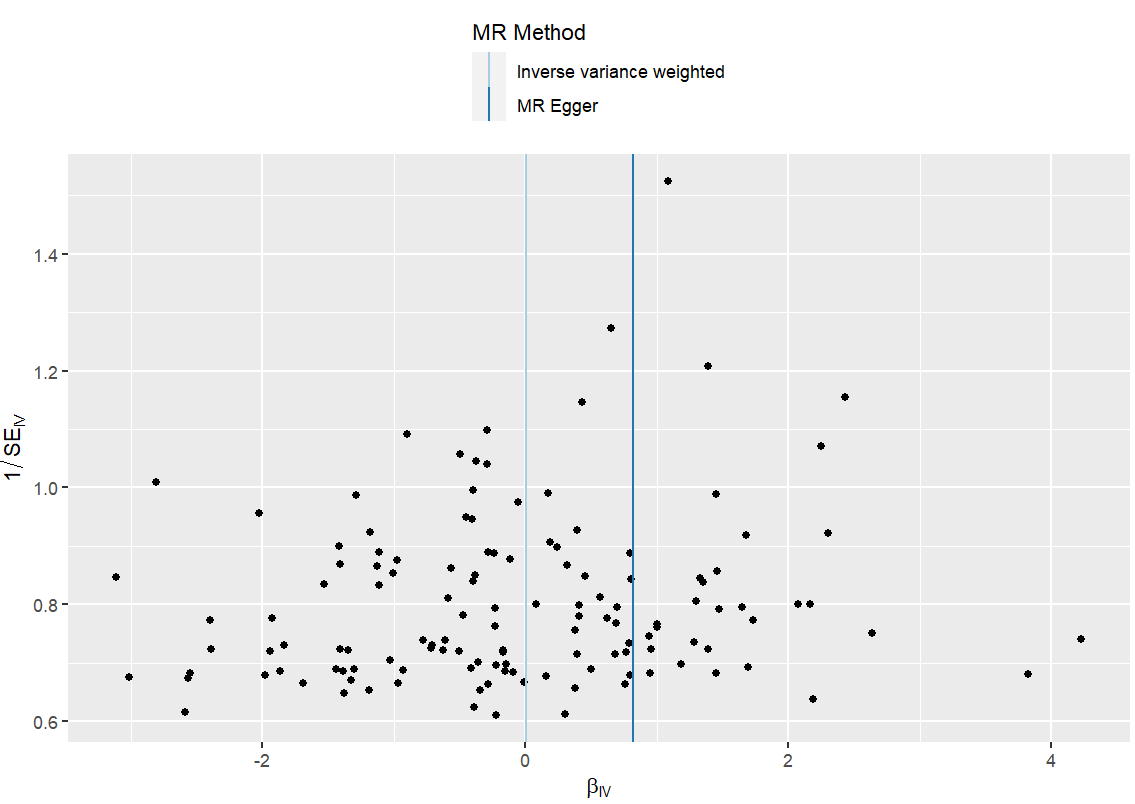


10. MR leave-one-out sensitivity analysis for Schizophrenia on Prostate cancer
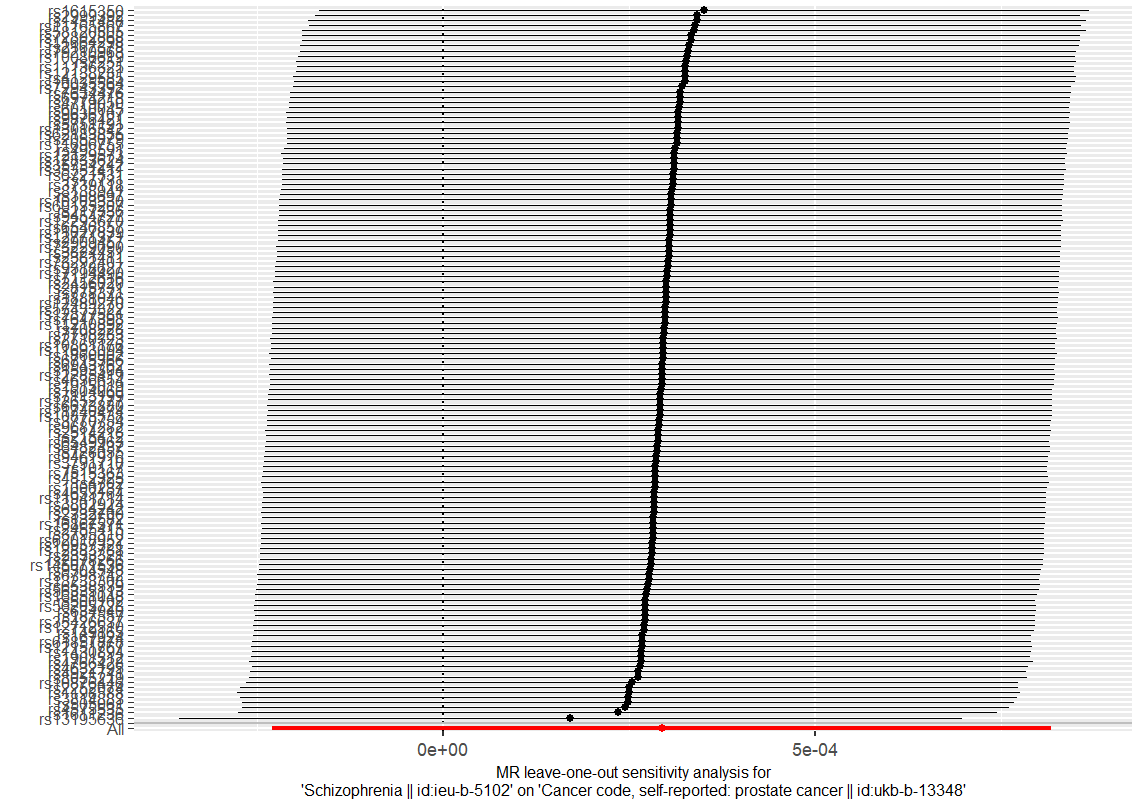


MR Scatter plot


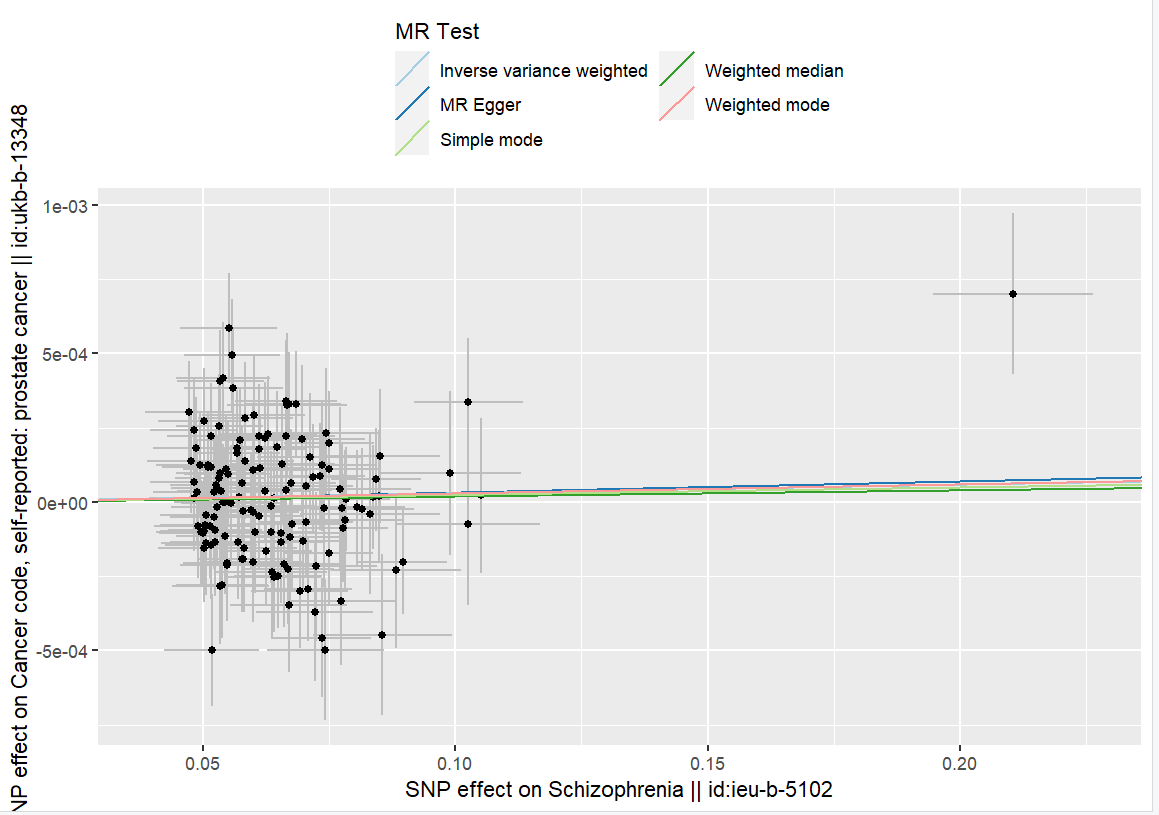


Forest map


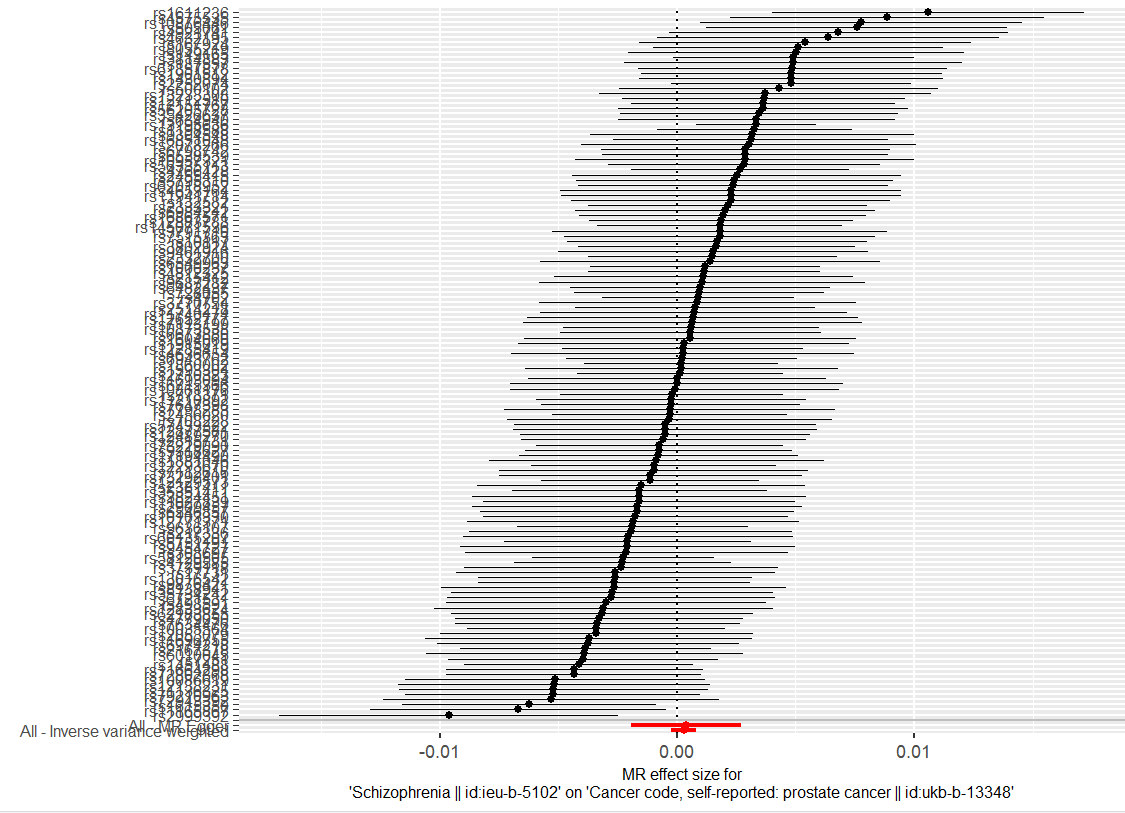


Funnel plot


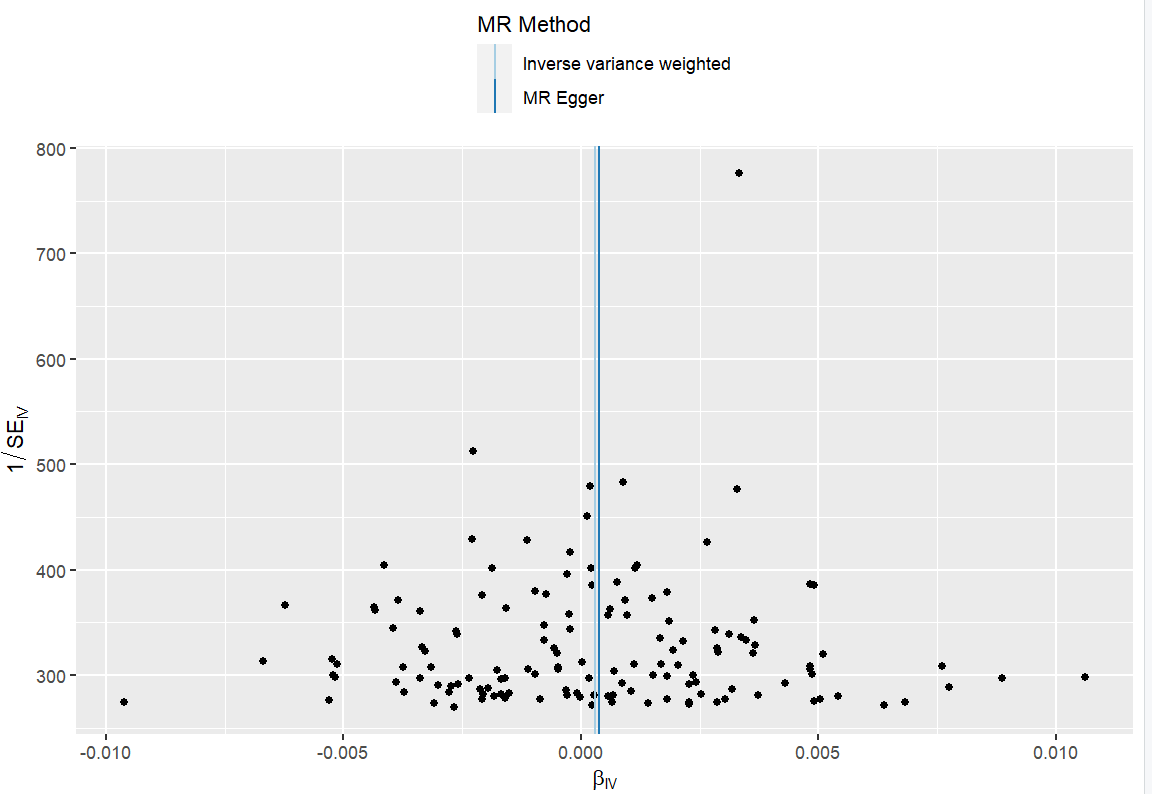


11. MR leave-one-out sensitivity analysis for Schizophrenia on Ovarian cancer
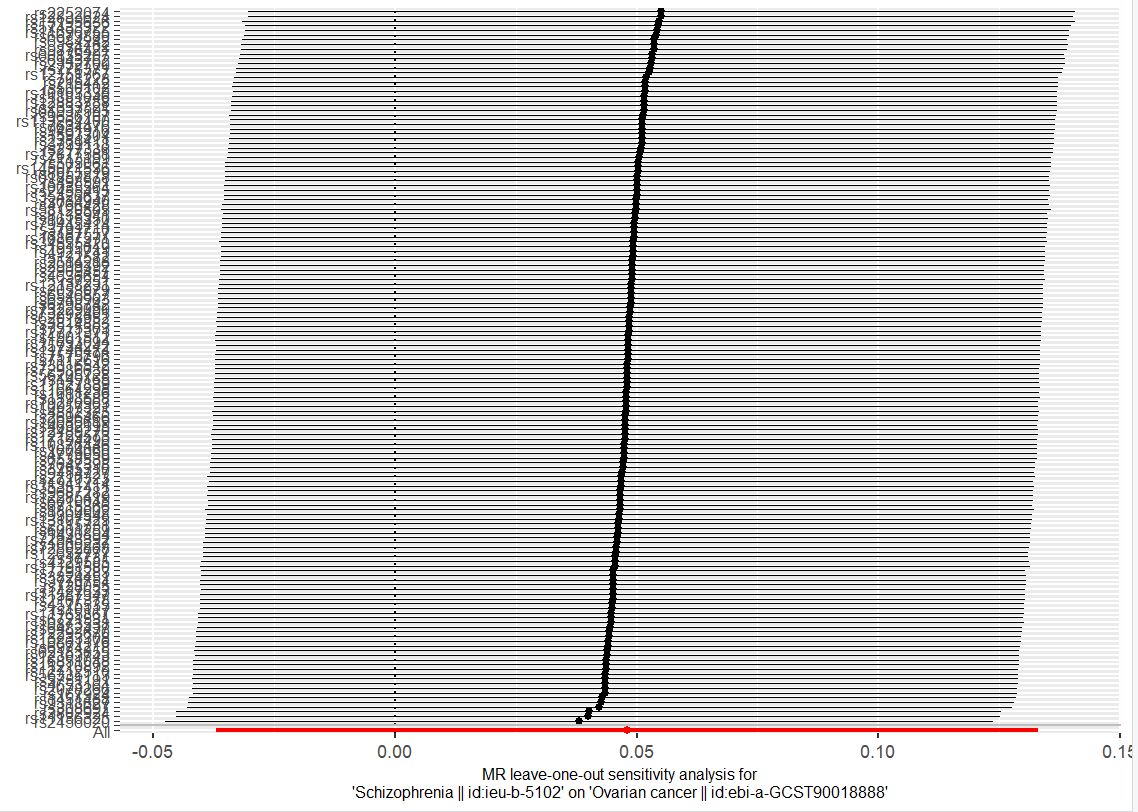


MR Scatter plot


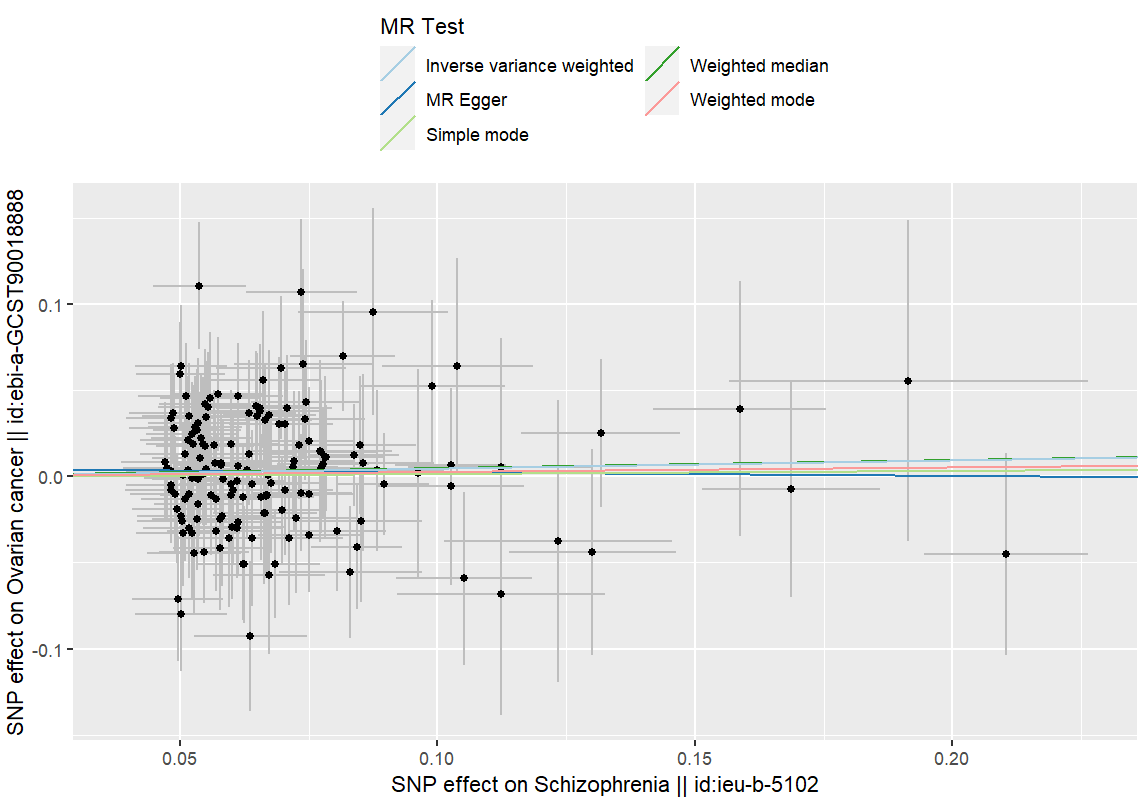


Forest map


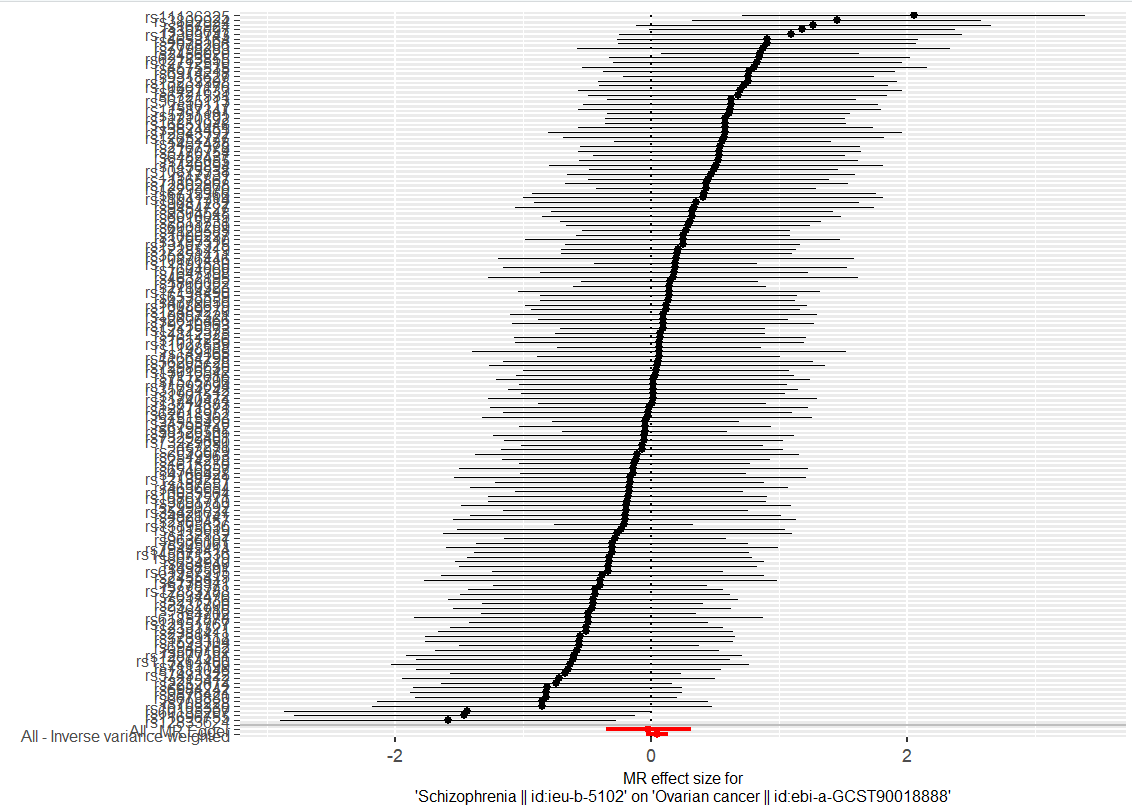


Funnel plot


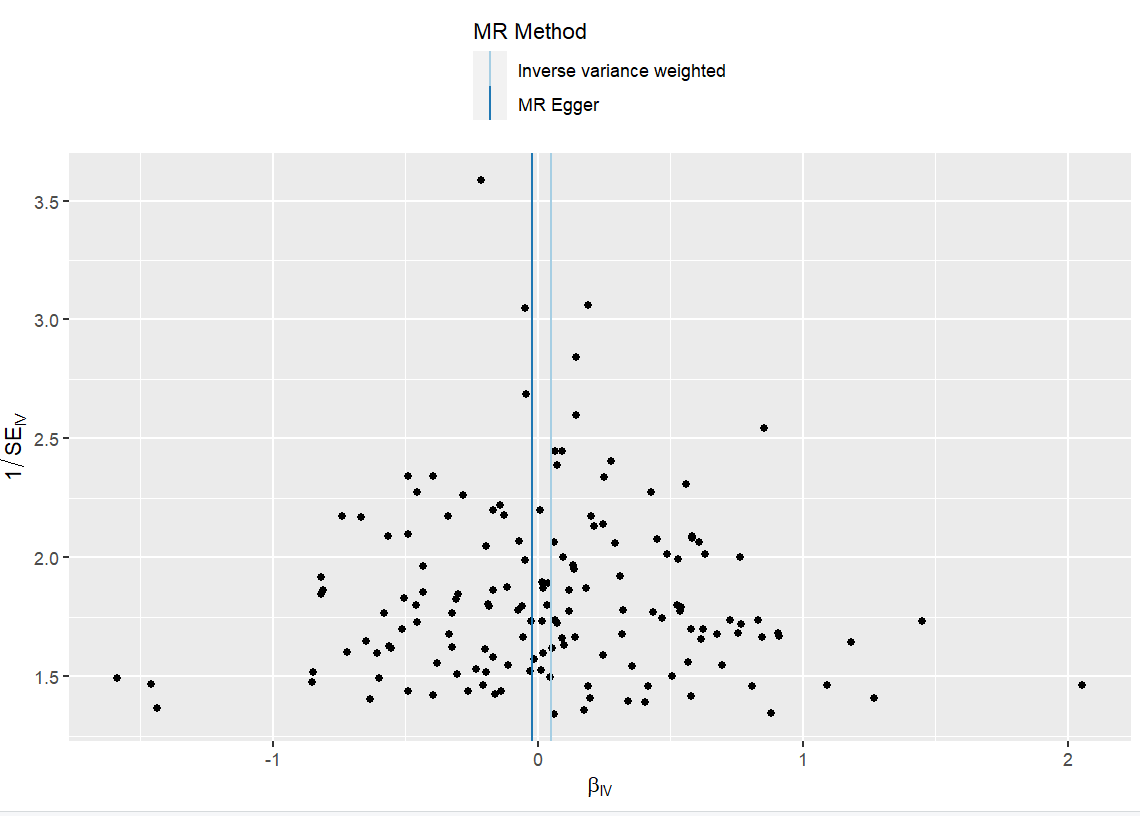


12. MR leave-one-out sensitivity analysis for Schizophrenia on Cervical cancer
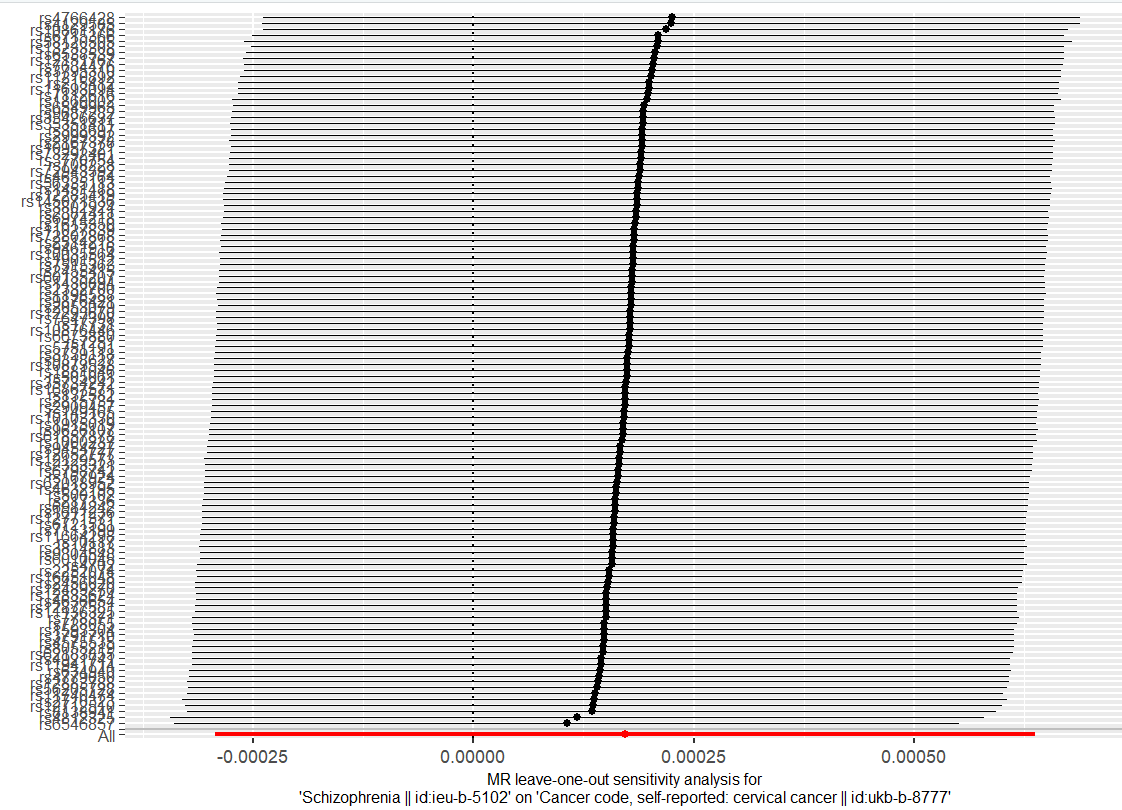


MR Scatter plot


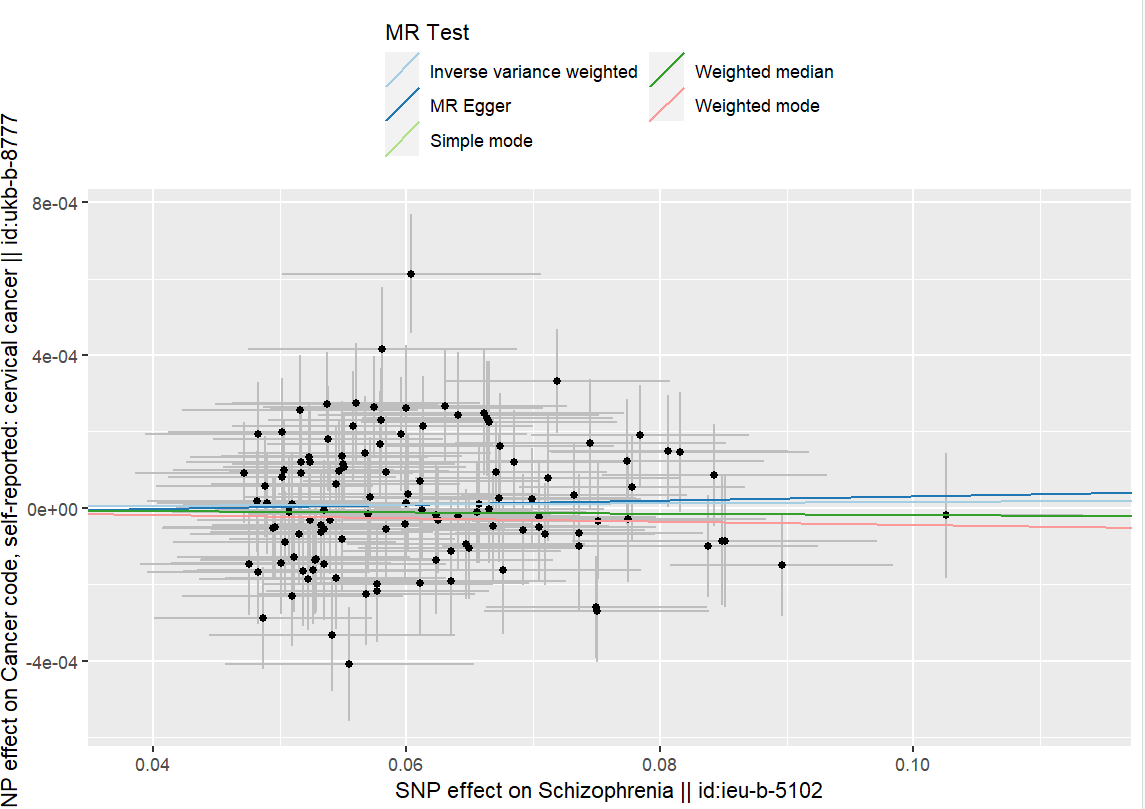


Forest map


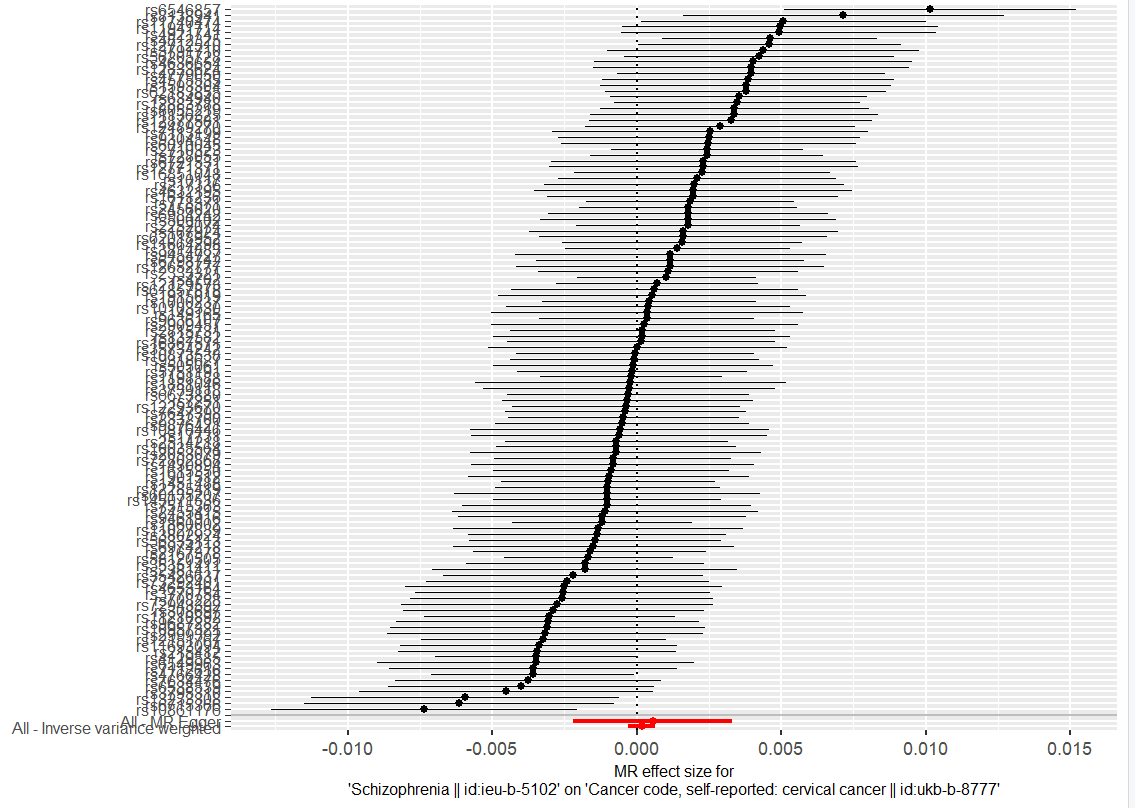


Funnel plot


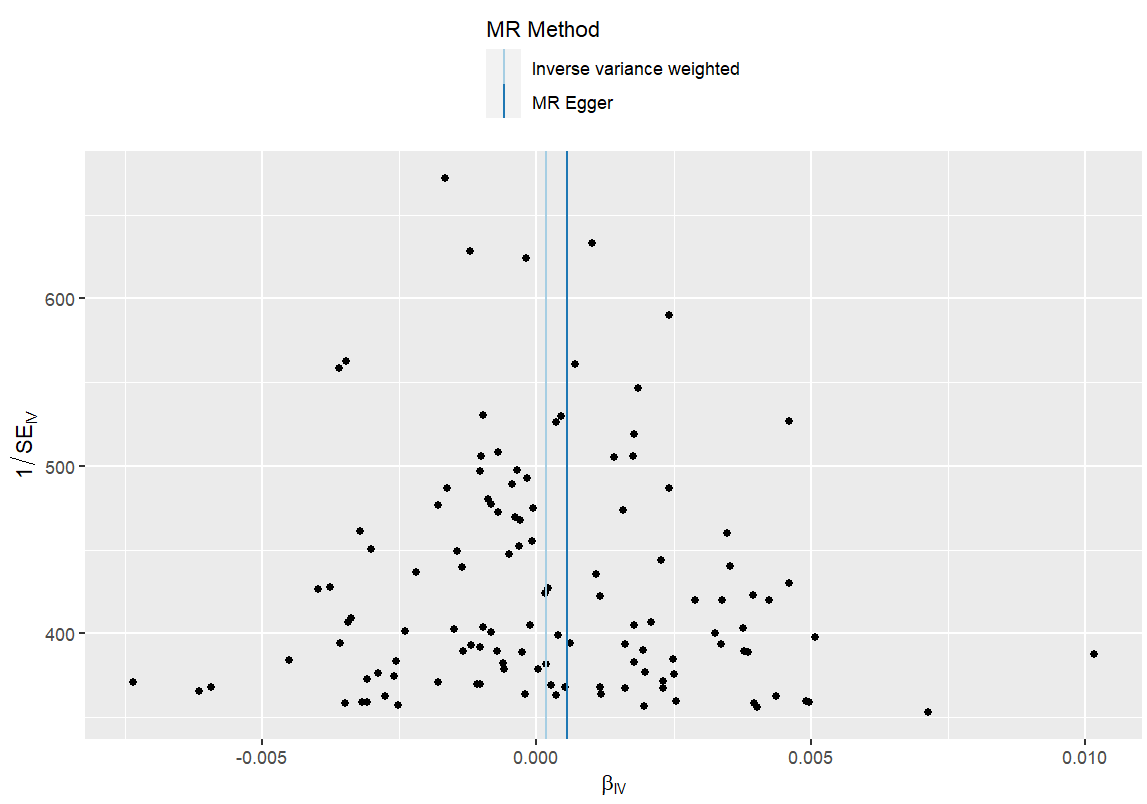


13. MR leave-one-out sensitivity analysis for Schizophrenia on Endometrial cancer
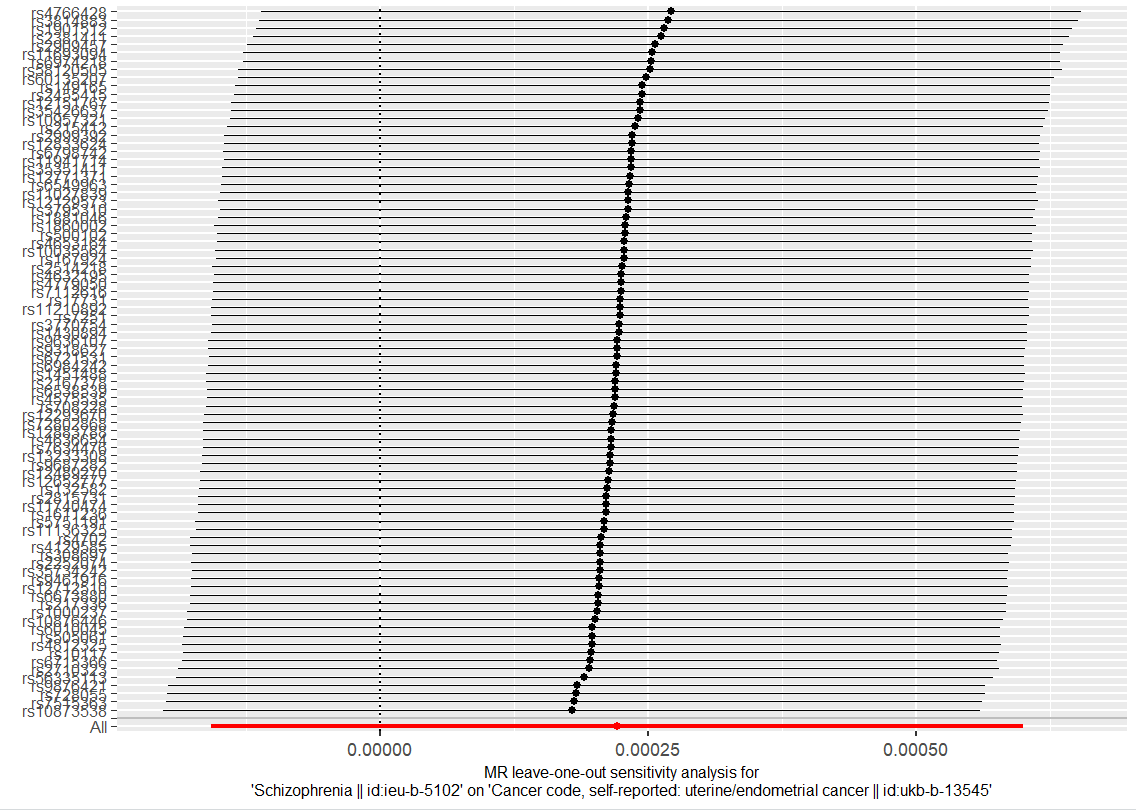


MR Scatter plot


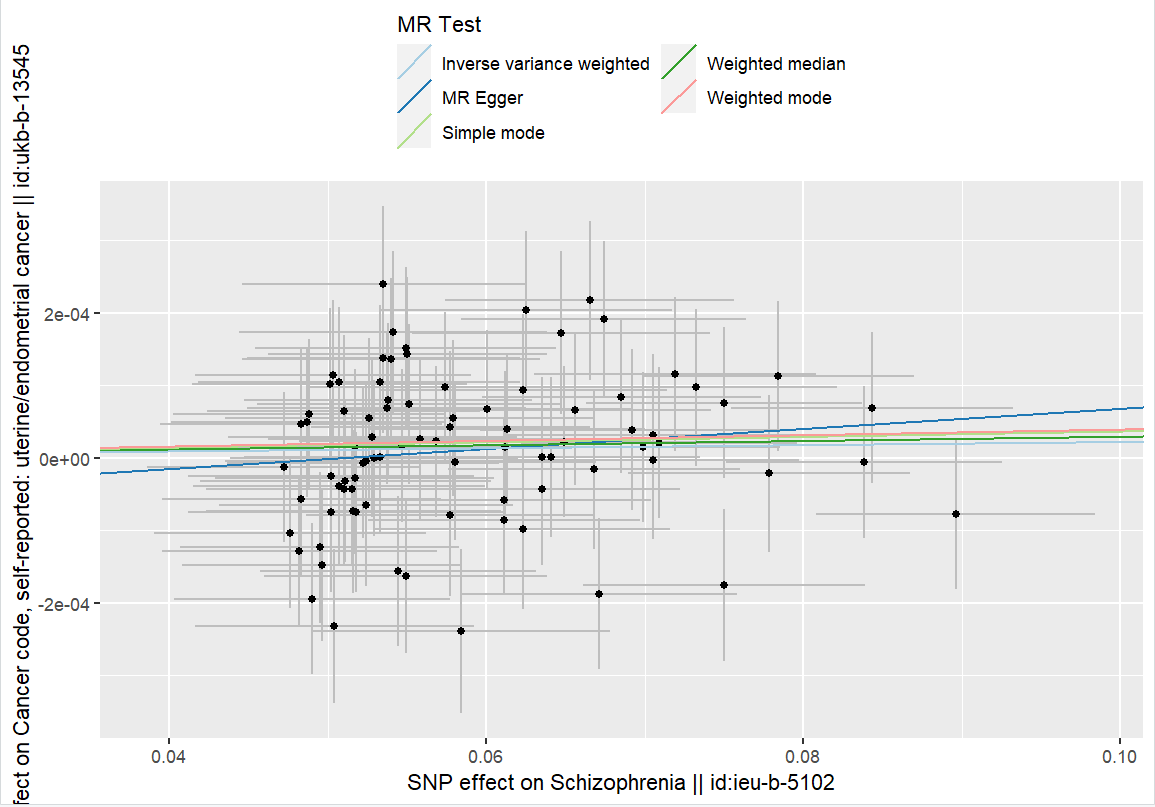


Forest map


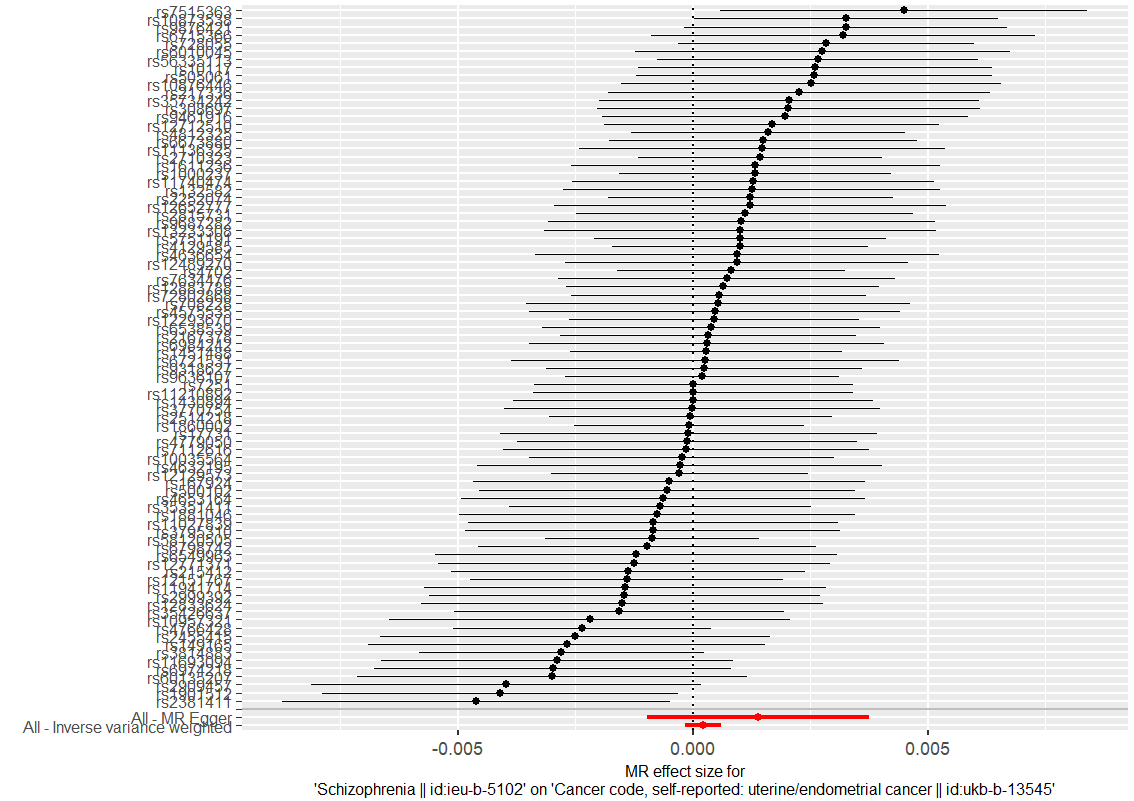


Funnel plot


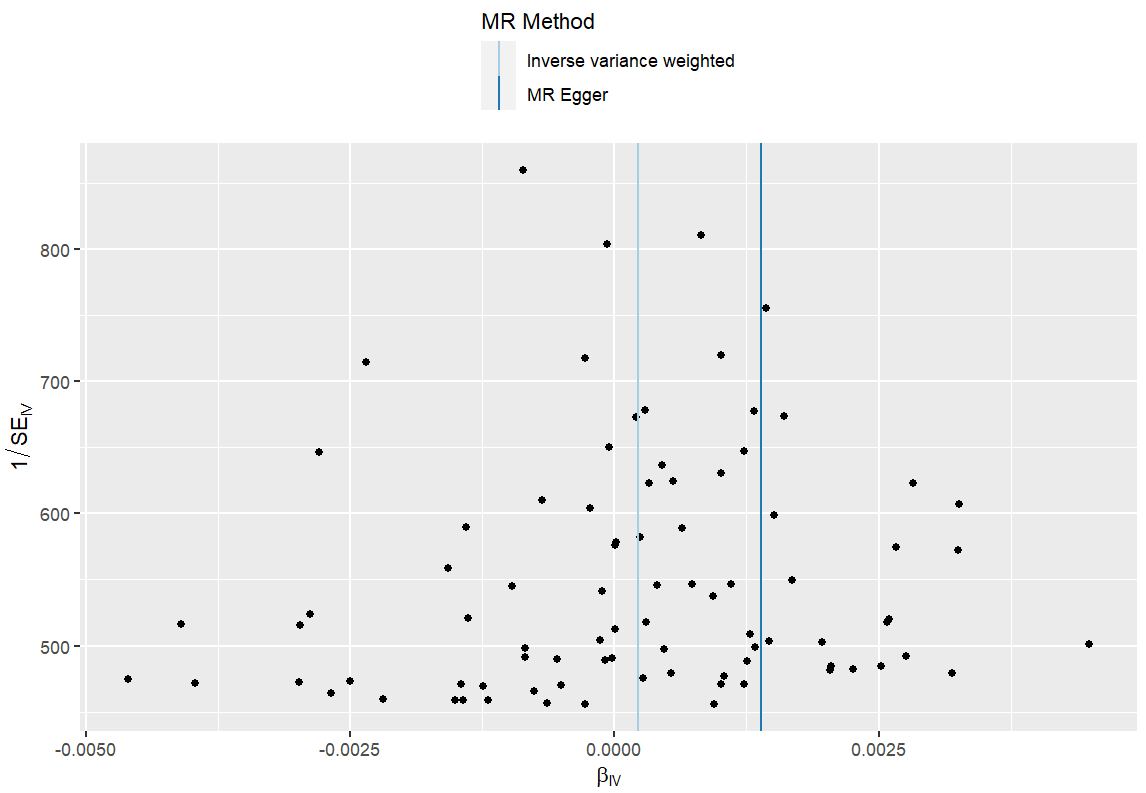


14. MR leave-one-out sensitivity analysis for Schizophrenia on Colon cancer
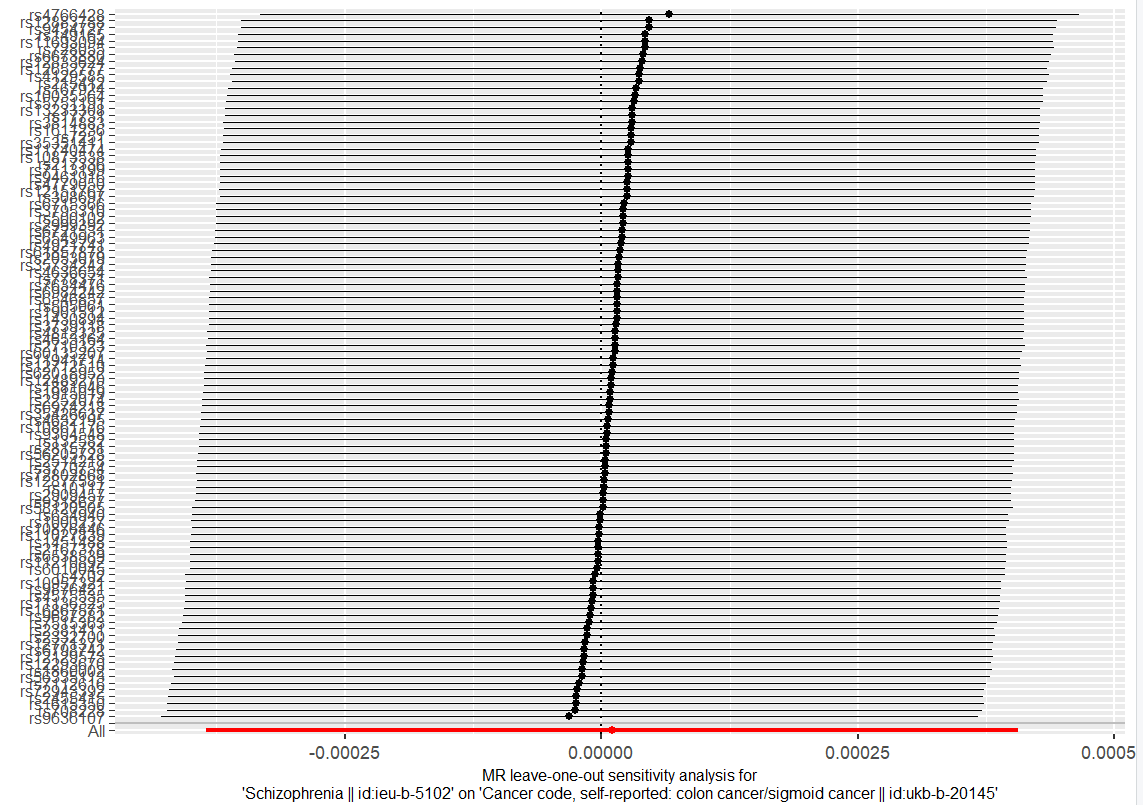


MR Scatter plot


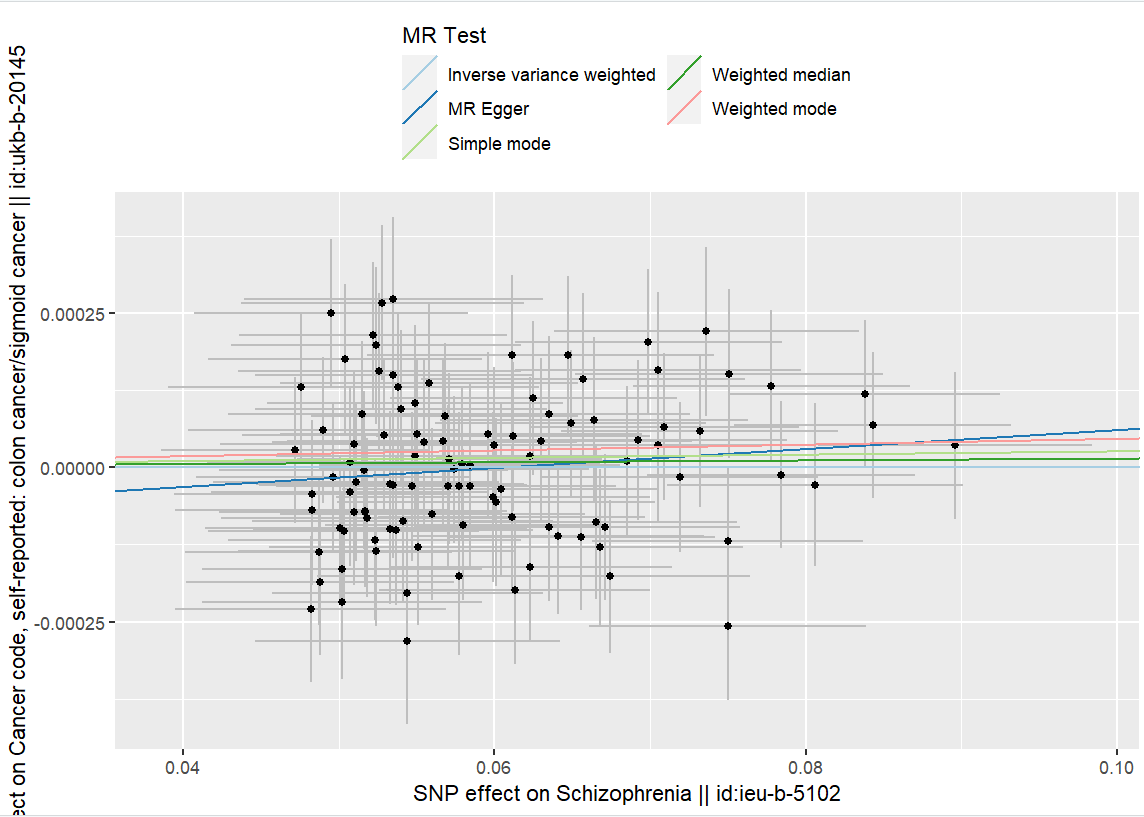


Forest map


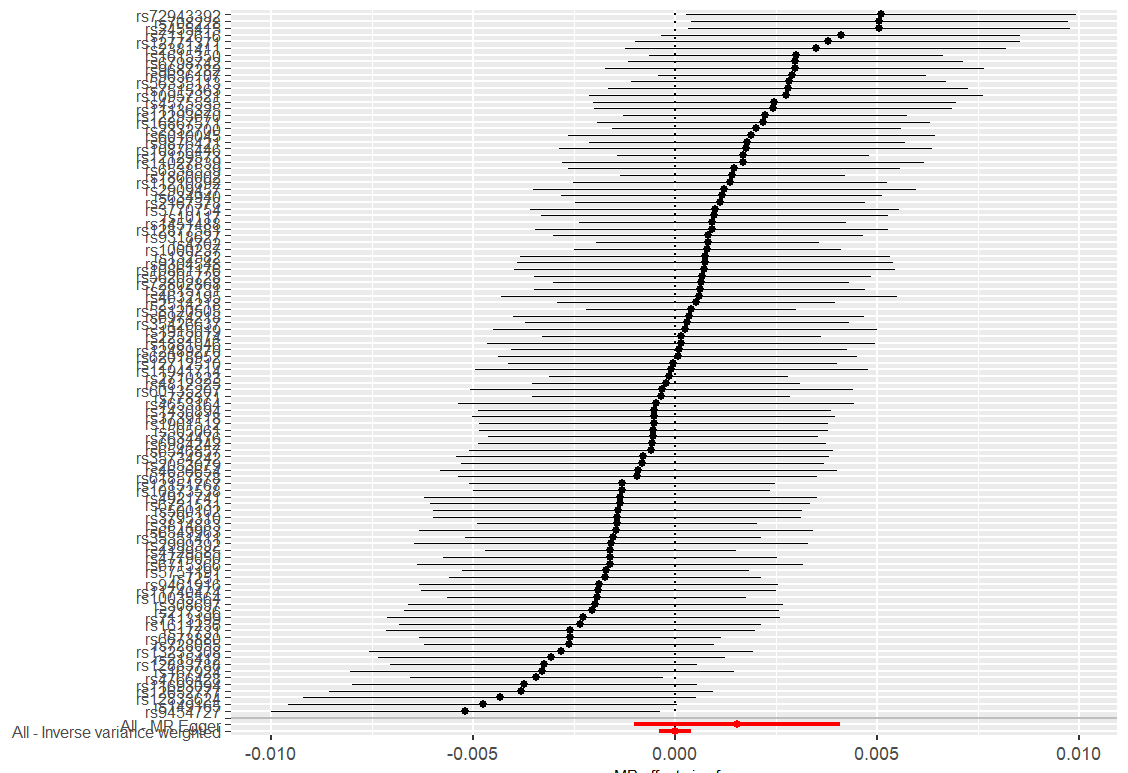


Funnel plot


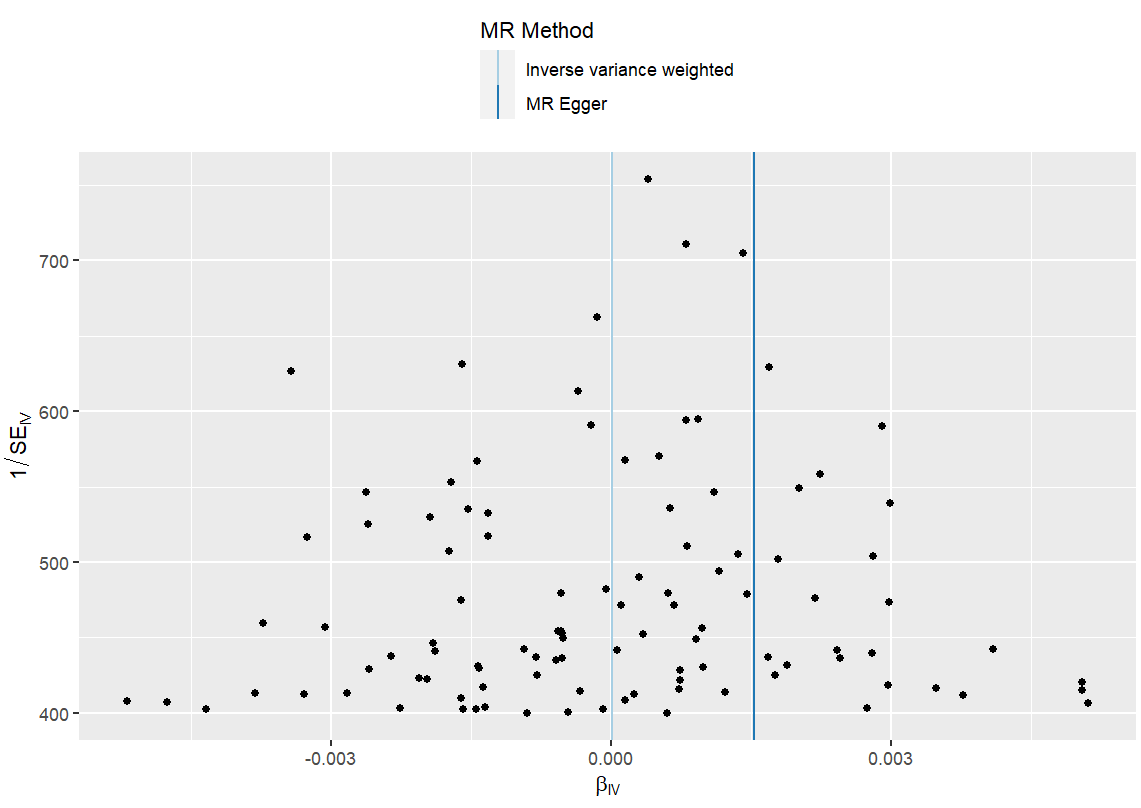


15. MR leave-one-out sensitivity analysis for Schizophrenia on Colorectal cancer
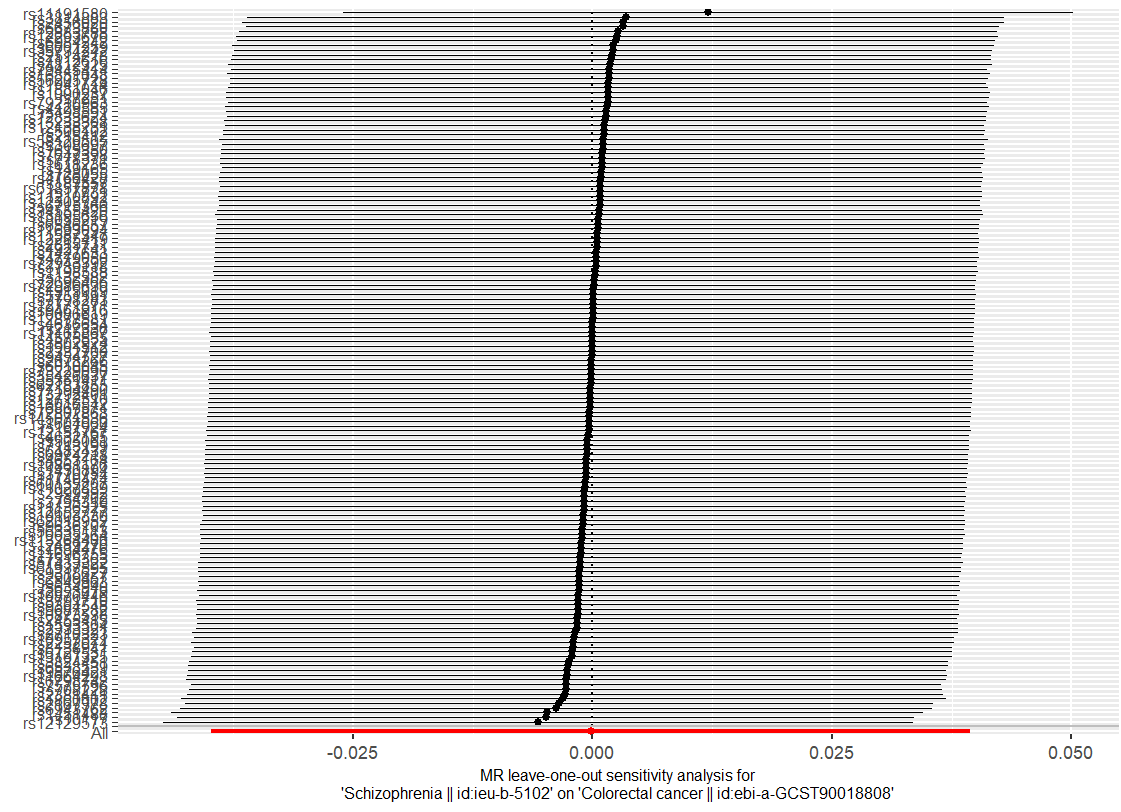


MR Scatter plot


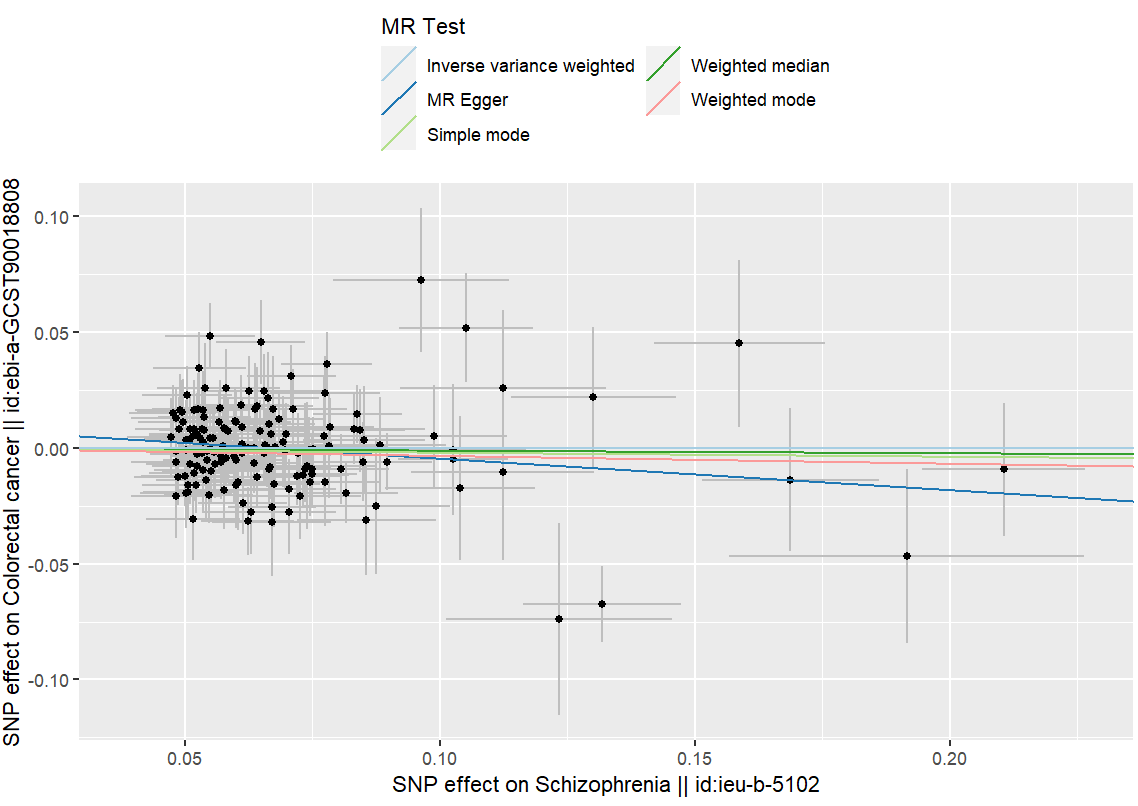


Forest map


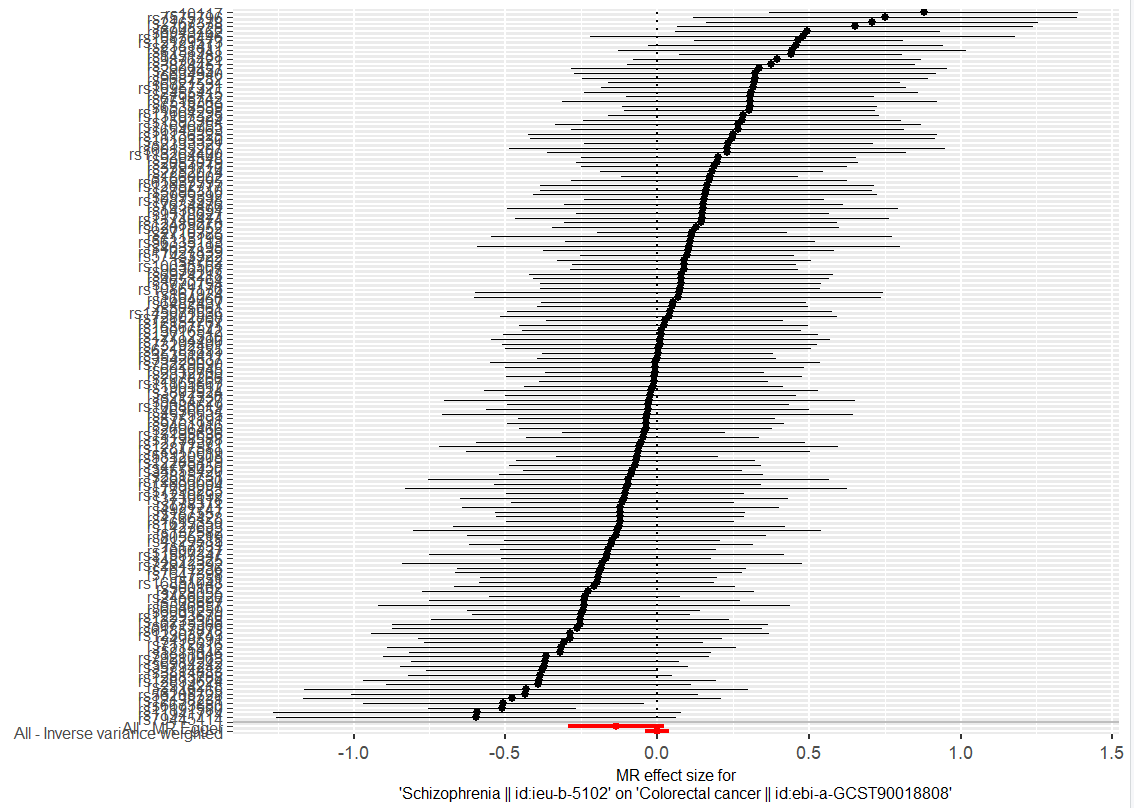


Funnel plot


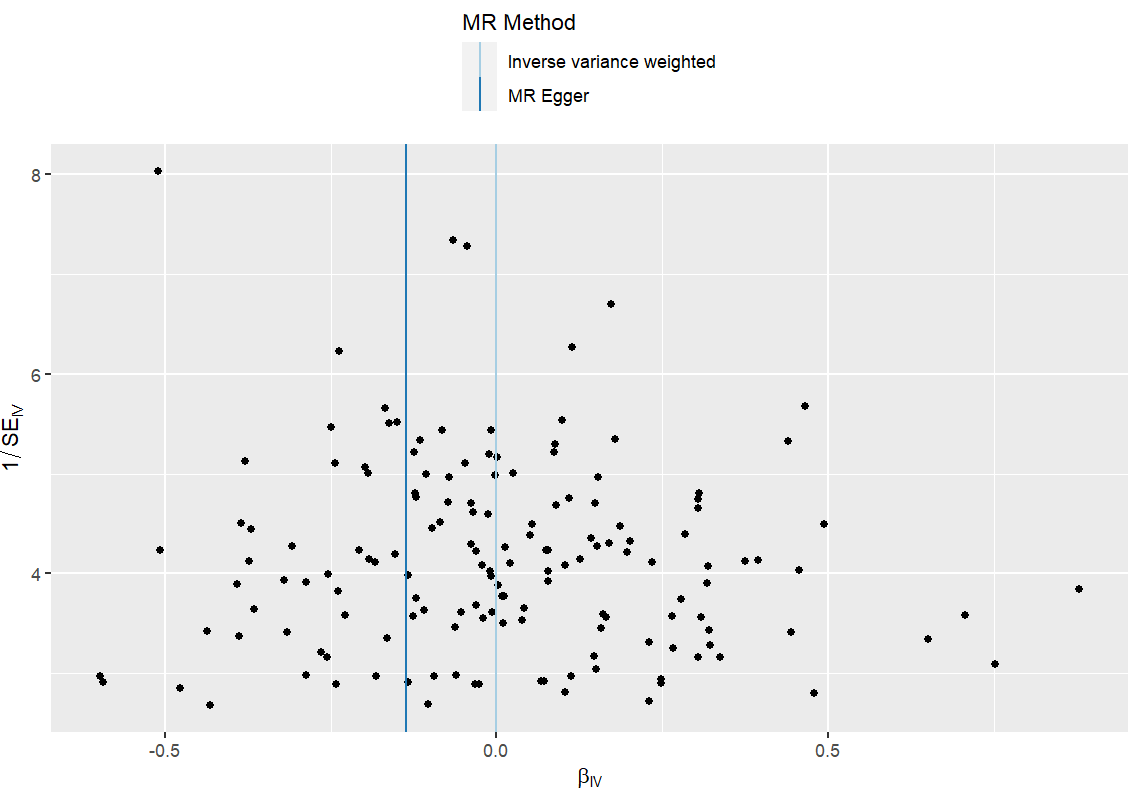


16. MR leave-one-out sensitivity analysis for Schizophrenia on Carcinoma of bladder
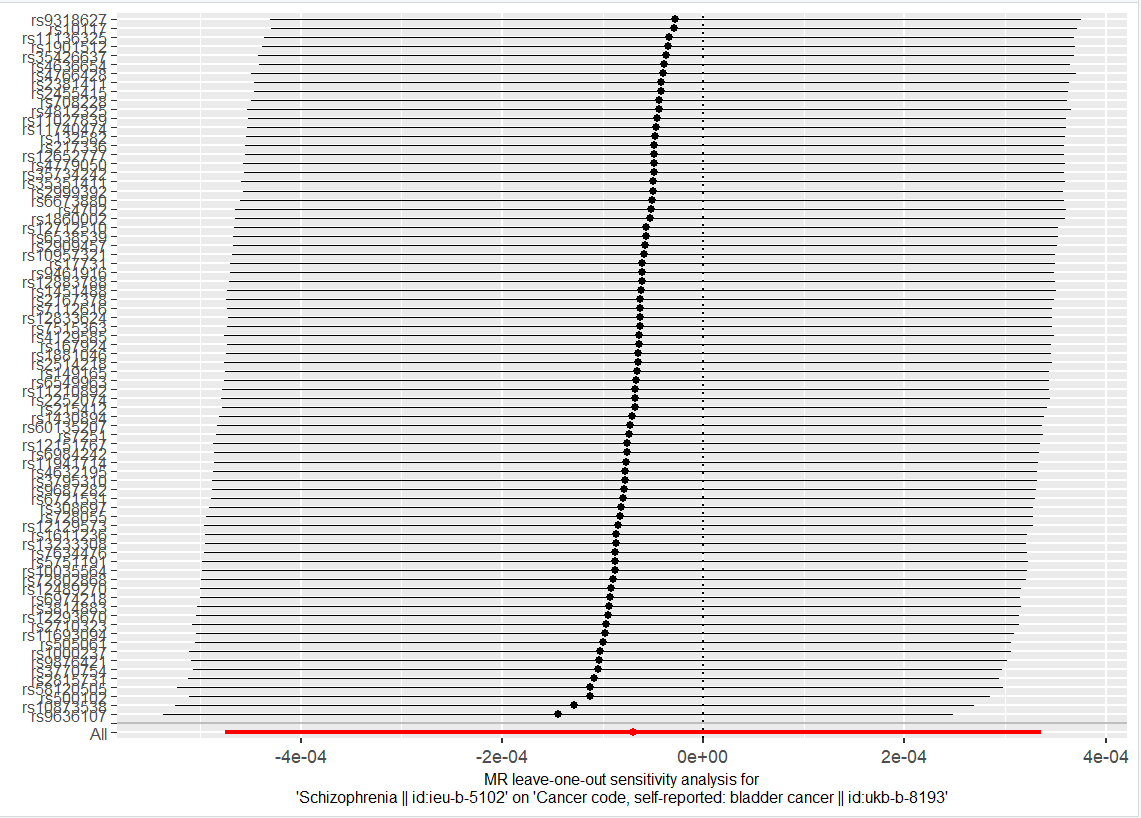


MR Scatter plot


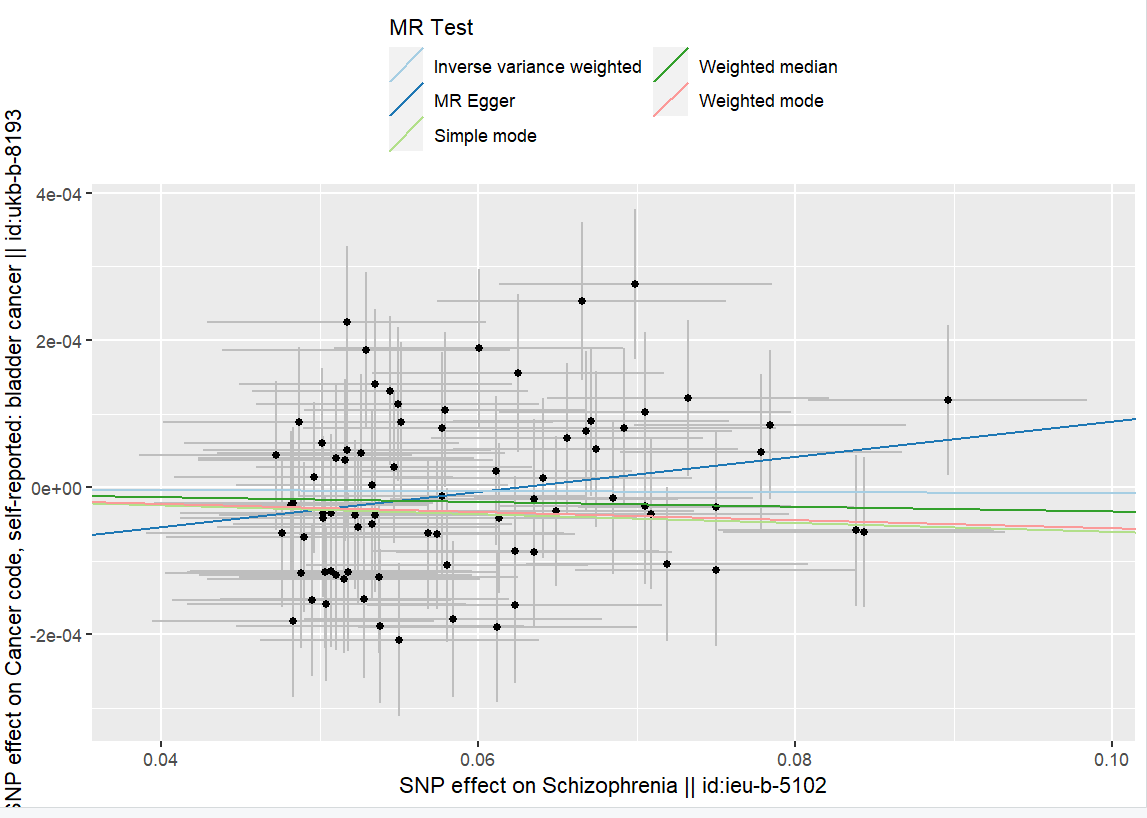


Forest map


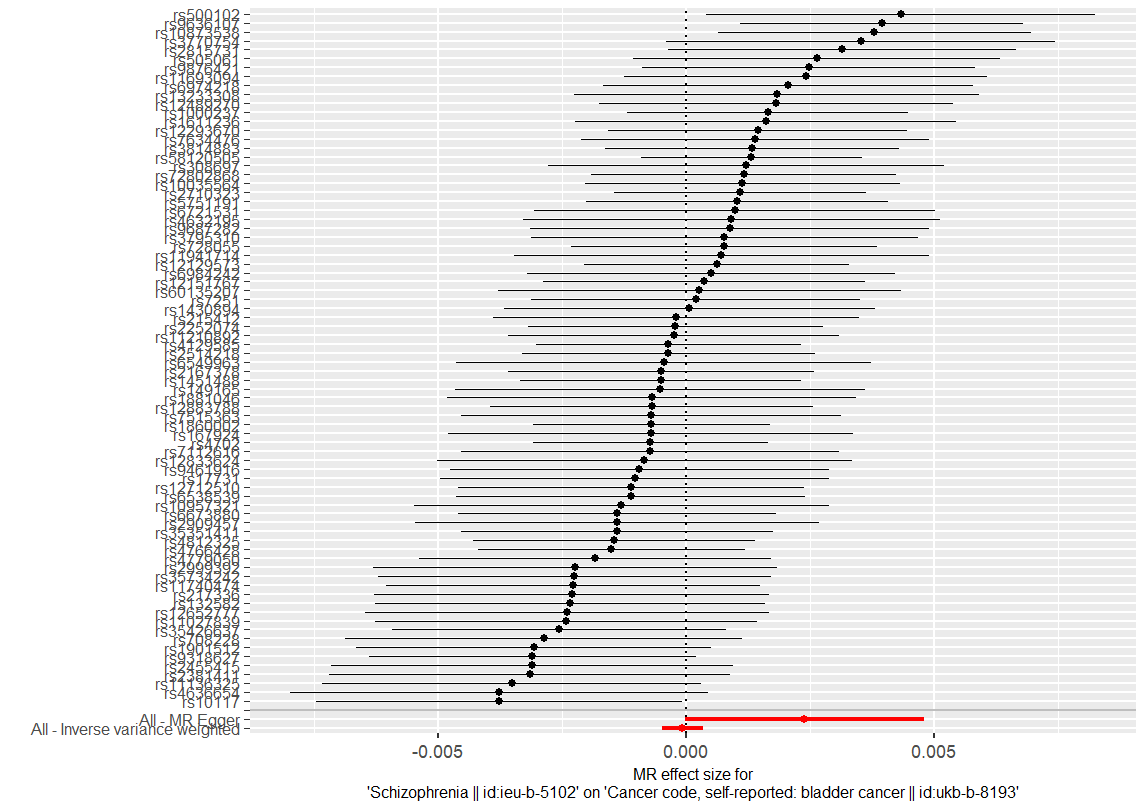


Funnel plot


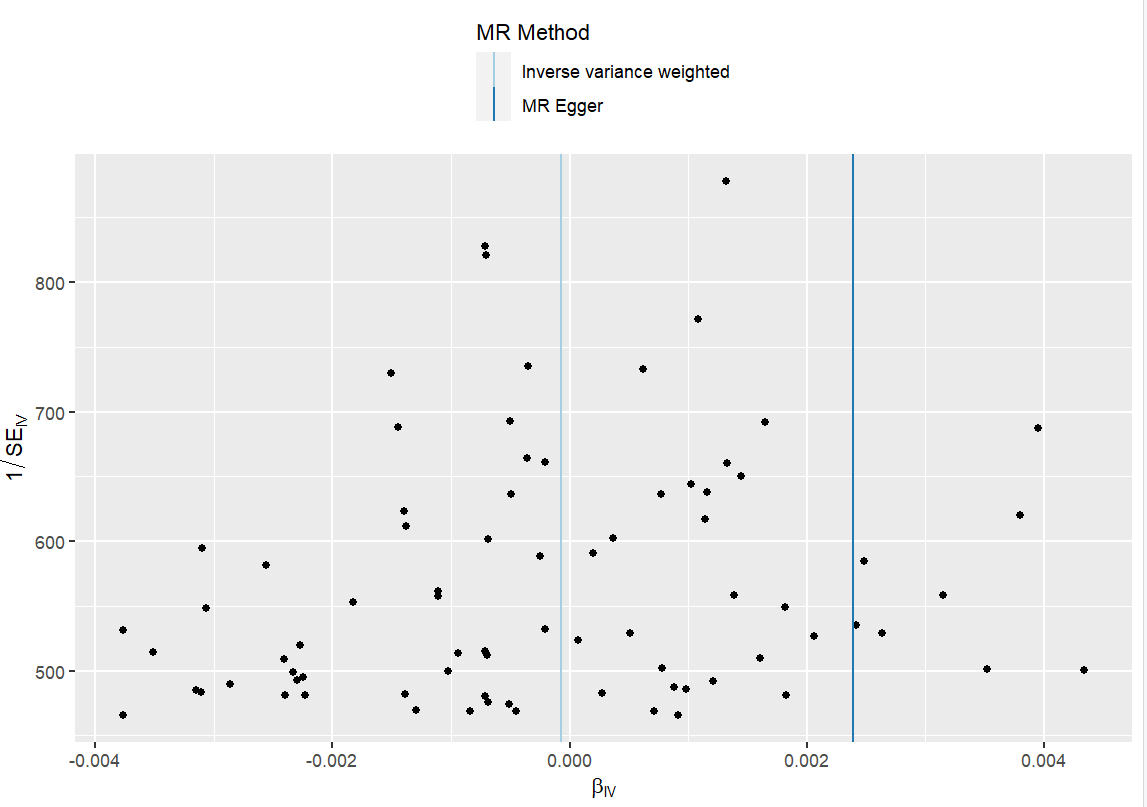

Supplement: Supplementary file 1 [file DataSheet_1.zip › supplementary materials/Figure.docx]
