# Supplementary figures and images for: Genetic prediction of the causal relationship between schizophrenia and tumors: a Mendelian randomized study
Source: Front Oncol. 2024 Feb 16;14:1321445. doi: 10.3389/fonc.2024.1321445 (PMC10905381; doi:10.3389/fonc.2024.1321445)

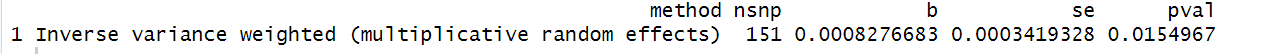

Supplement: Supplementary file 1 [file DataSheet_1.zip › supplementary materials/Random-efect method.png]
